# Cost-effectiveness of Prostate Cancer Screening Using Magnetic Resonance Imaging or Standard Biopsy Based on the STHLM3-MRI Study

**DOI:** 10.1001/jamaoncol.2022.5252

**Published:** 2022-11-10

**Authors:** Shuang Hao, Andrea Discacciati, Martin Eklund, Emelie Heintz, Ellinor Östensson, K. Miriam Elfström, Mark S. Clements, Tobias Nordström

**Affiliations:** 1 Department of Medical Epidemiology and Biostatistics, Karolinska Institutet, Stockholm, Sweden; 2 Department of Learning, Informatics, Management and Ethics, Karolinska Institutet, Stockholm, Sweden; 3 Department of Laboratory Medicine, Karolinska Institutet, Stockholm, Sweden; 4 Department of Clinical Sciences, Danderyd Hospital, Danderyd, Sweden

## Abstract

**Importance:**

The combination of prostate-specific antigen (PSA) testing with magnetic resonance imaging (MRI) for prostate cancer detection has rarely been evaluated in a screening context. The STHLM3-MRI screening-by-invitation study (NCT03377881) has reported the benefits of using MRI with subsequent combined targeted and standard biopsies compared with using standard biopsies alone.

**Objective:**

To investigate the cost-effectiveness of prostate cancer screening using MRI with combined targeted and standard biopsies compared with standard biopsies alone among men aged 55 to 69 years in Sweden, based on evidence from the STHLM3-MRI study.

**Design, Setting, and Participants:**

This economic evaluation study was conducted from a lifetime health care perspective using a microsimulation model to evaluate no screening and screening strategies among adult men in Sweden. Men aged 55 to 69 years in Sweden were simulated for no screening and screening strategies. Input parameters were obtained from the STHLM3-MRI study and recent reviews. One-way and probabilistic sensitivity analyses were performed in May 2022.

**Interventions:**

No screening, quadrennial PSA screening using standard biopsies alone, and MRI-based screening using combined targeted and standard biopsies.

**Main Outcomes and Measures:**

The number of tests, incidence, deaths, costs, quality-adjusted life-years (QALY), and incremental cost-effectiveness ratios (ICERs) were estimated.

**Results:**

A total 603 men were randomized to the standard arm, 165 of these participants (27.4%) did not undergo standard biopsy; 929 men were randomized to the experimental arm, 111 (11.9%) of whom did undergo MRI or any biopsy. Compared with no screening, the screening strategies were associated with reduced lifetime prostate cancer–related deaths by 6% to 9%. Screening with MRI and the combined biopsies resulted in an ICER of US $53 736, which is classified as a moderate cost per QALY gained in Sweden. Relative to screening with standard biopsies alone, MRI-based screening reduced the number of both lifetime biopsies and overdiagnosis by approximately 50% and had a high probability of being cost-effective than the traditional PSA screening.

**Conclusions and Relevance:**

For prostate cancer screening, this economic evaluation study found that PSA testing followed by MRI with subsequent combined targeted and standard biopsies had a high probability to be more cost-effective compared with the traditional screening pathway using PSA and standard biopsy. MRI-based screening may be considered for early detection of prostate cancer in Sweden.

## Key Points

**Question:**

Within a prostate cancer screening context, is magnetic resonance imaging (MRI)-based screening more cost-effective than no screening and screening using prostate-specific antigen (PSA) alone?

**Findings:**

In this economic evaluation study of 1532 adult men in Sweden, cost-effectiveness analysis showed that MRI-based screening with combined targeted and standard biopsies was associated with reduced lifetime prostate cancer–related deaths and resulted in an incremental cost-effectiveness ratio of US $53 736 per quality-adjusted life-year gained compared with no screening. Compared with PSA screening, MRI-based screening reduced the number of both lifetime biopsies and overdiagnosis by approximately 50%.

**Meaning:**

These results suggest that screening with PSA and MRI with subsequent combined biopsies for prostate cancer has a high probability to be more cost-effective compared with PSA screening using standard biopsy.

## Teaser

This economic evaluation study of Swedish men compares the cost-effectiveness of magnetic resonance imaging–aided screening for prostate cancer with strategies using no screening or prostate-specific antigen alone.

## Introduction

Screening using the prostate-specific antigen (PSA) test followed by a standard biopsy has been found to reduce prostate cancer mortality.^1^ However, organized screening remains debatable due to the possibility of unnecessary biopsies with associated adverse effects as well as the high possibility of overdiagnosis and overtreatment of clinically insignificant cancers. In recent years, there has been growing evidence in favor of magnetic resonance imaging (MRI) combined with targeted biopsies to complement PSA testing and improve prostate cancer diagnostics.^2,3,4^

The test characteristics of using MRI with combined targeted and standard biopsies in men receiving positive MRI tests for prostate cancer diagnosis were recently reviewed by the Cochrane Collaboration. Importantly, the studies from the Cochrane Review found that 30% of men had a negative MRI finding,^5^ while the Göteborg 2 screening trial found that 75% of men had a negative MRI finding.^6^ This contrast exhibits an important difference by the study setting, where far fewer of the patients from a diagnostic setting, as per the Cochrane Review, had a negative MRI result compared with findings from a screening-by-invitation setting.^6^

The STHLM3-MRI study was a Swedish population-based screening-by-invitation trial for men aged 50 to 74 years (ClinicalTrials.gov Identifier, NCT03377881).^3,7^ Participants with PSA levels at 3 ng/mL or higher (to convert to micrograms per liter, multiply by 1.0) were randomly assigned to undertake a 10- to 12-core standard biopsy in the standard arm, or undergo a triage test of MRI and a combined MRI-guided targeted biopsy and standard biopsy in the experimental arm if the MRI had a Prostate Imaging Reporting and Data System (PI-RADS) value between 3 and 5.^3^ Using an intention-to-treat analysis, the trial found that the experimental arm was noninferior for detecting clinically significant cancers compared with the standard arm (21% vs 18%), with fewer clinically insignificant cancers in the experimental arm (4% vs 12%).^3^ The results from this trial provided level 1 evidence on the effectiveness of MRI and combined biopsy approach for detecting prostate cancer in a screening setting. Using results from the STHLM3-MRI study, our aim was to assess the cost-effectiveness of quadrennial PSA-based prostate cancer screening using MRI and a combined biopsy approach in men aged 55 to 69 years in Sweden.

## Methods

### Analytical Approach

The most commonly used approach to assess cost-effectiveness of cancer screening is simulating for the disease natural history.^8^ Such an approach combines the epidemiological evidence from health and population registers, information on costs and health state values, and evidence from the randomized controlled trials or diagnostic trials. It addresses the common issues that cancer screening trials are large, expensive, and may take 10 to 20 years to provide reliable estimates of effectiveness. In this study, we proposed a cost-utility analysis using a microsimulation model to assess the cost-effectiveness of different strategies. For reporting, we followed the Consolidated Health Economic Evaluation Reporting Standards (CHEERS) 2022 checklist.^9^ All data were analyzed anonymously. The regional Swedish ethical review board of Stockholm approved the use of the data from the Stockholm PSA and Biopsy Register without informed consent, and from the STHLM3-MRI Study with written informed consent.

### Study Population and Screening Strategies

**Figure 1.**
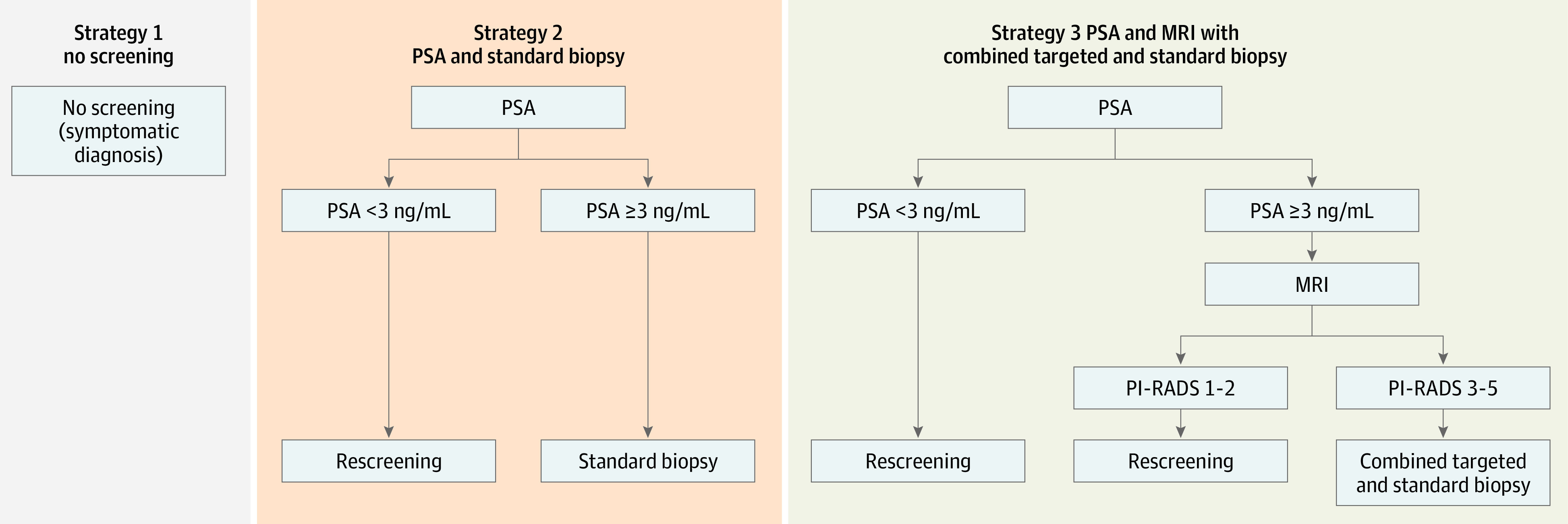
Illustrations of Strategies MRI indicates magnetic resonance imaging; PI-RADS, Prostate Imaging–Reporting and Data System; PSA, prostate-specific antigen.

We modeled for no screening (strategy 1) and 2 screening strategies: standard biopsies (strategy 2) and MRI with combined targeted and standard biopsies for men who have PI-RADS values between 3 and 5 (strategy 3) (Figure 1). For strategy 1, we assumed prostate cancer was only diagnosed for men with symptoms. Following the European Randomized Screening of Prostate Cancer (ERSPC) trial,^10^ men aged 55 to 69 years without a diagnosis of prostate cancer were modeled in both quadrennial PSA-based screening strategies and were assumed to be administered by general practitioners with referral to a specialist for PSA levels of 3 ng/mL or higher.

### Simulation Model

An open-source microsimulation model was used to simulate the life histories of individuals, including prostate cancer testing, cancer onset, progression, diagnosis, disease management, and deaths^11^ (eFigure 1 in the Supplement). The biopsy and treatment pathways were informed by data from the Stockholm PSA and Biopsy Register (SPBR).^12^ The survival for M0 cancers by age and International Society of Urological Pathology (ISUP) grade group and for M1 cancers by age was retrieved from the National Prostate Cancer Register.^13^ The model was calibrated to the incidence rate ratio from ERSPC and prostate cancer incidence in Sweden.^11^ Outcomes and costs for all strategies, including no screening, were modeled through the calibrated natural history model. The model allows progression between preclinical T-stages to metastasis.^11^ Details of the natural history model were provided by Karlsson et al.^11^

### Test Characteristics

The per-protocol test characteristics were estimated using multiple imputation based on the data from the standard and experimental arms by the STHLM3-MRI study.^3^ We conducted model-based multiple imputations for benign biopsies, grade group 1 cancers (ie, clinically insignificant), and cancers in grade groups 2 and above (clinically significant) for (1) the standard arm, with the 165 of 603 participants (27.4%) who had PSA levels above 3 ng/mL but who did not undergo standard biopsies cases, and (2) the experimental arm, where 111 out of 929 participants (11.9%) did not follow the study protocol. Using the imputed data, the relative positive fractions of benign biopsies, grade group 1, and grade group 2 or higher cancers were calculated to compare MRI and combined biopsies with standard biopsies (eMethods in the Supplement). Because participants with negative MRI results did not undertake biopsies per protocol in the experimental arm, an adjustment was conducted to estimate the number of cases by grading. As the test performance estimated using a screening-by-invitation setting might differ from a diagnostic setting, the log-relative positive fractions of the estimates based on the STHLM3-MRI trial were compared with the estimates from our meta-analysis based on the data from the Cochrane Review using a χ^2^ test.

The estimated test characteristics included: the probability of a positive MRI result given benign biopsies, grade group 1, or grade group 2 or higher cancers; and the false negative rate of using PSA and standard biopsies given grade group 1 or grade group 2 or higher cancers (Table 1). For the false negative rates, we assumed that the maximum ISUP grade group from either targeted or standard biopsies was the reference. We assumed a 75% and 95% attendance for screening and rescreening, respectively, and 85.6% biopsy compliance.^1^

**Table 1.** Input Parameters Used in the Cost-effectiveness Analysis

Test characteristics^a^	Value (95% CI)
Pr(MRI+ | PSA ≥3, benign biopsies, MRI and combined biopsies)	0.184 (0.147-0.229)
Pr(MRI+ | PSA ≥3, grade group 1, MRI and combined biopsies)	0.317 (0.198-0.465)
Pr(MRI+ | PSA ≥3, grade group ≥2, MRI and combined biopsies)	0.837 (0.643-0.936)
Pr(SBx− | PSA ≥3, grade group 1, standard biopsy)	0.063 (0.028-0.135)
Pr(SBx− | PSA ≥3, grade group ≥2, standard biopsy)	0.099 (0.064-0.151)
Pr(TBx− | PSA ≥3, grade group 1, MRI and targeted biopsy)	0.543 (0.366-0.712)
Pr(TBx− | PSA ≥3, grade group ≥2, MRI and targeted biopsy)	0.062 (0.031-0.108)

Abbreviations: MRI, magnetic resonance imaging; PSA, prostate-specific antigen; SBx, standard biopsy; TBx, targeted biopsy.

^a^ Formulae for test characteristics test the probability (Pr) of an outcome (ie, a positive MRI test) given the variables that follow. Source used for test characteristics was the STHLM3-MRI study.^3^

### Reported Outcomes

For each strategy, we modeled the mean lifetime number of screening tests, MRIs, biopsies, incidence, incidence under screening ages 55 to 69 years, overdiagnosed cases, deaths, and life expectancy for a cohort of 10 million men followed from age 55 years. Overdiagnosis was defined as those who were detected by screening but would never have presented symptoms before deaths due to other causes.

### Cost-effectiveness Analysis

Our base-case analysis was conducted from a health care perspective using a lifetime horizon.^14^ We also reported the results from a societal perspective. As per Drummond et al,^15^ the strategies were ranked from low to high costs. The incremental cost-effectiveness ratio (ICER) was calculated by dividing the difference in costs by the difference in quality-adjusted life-years (QALYs) between 2 strategies. The results were presented in Swedish kronor (SEK) and US dollars (US $) using the average exchange rate of $1 equaling 9.2037 SEK from 2020.^16^ We applied the cost-effectiveness categories defined by the National Board of Health and Welfare, which included costs below 100 000 SEK (US $10 856), between 100 000 and 499 999 SEK (US $10 856-$54 326), between 500 000-1 million SEK (US $54 326-$108 652), and costs above 1 million SEK (US $108 652) per QALY gained as low, moderate, high, and very high costs, respectively.^17^ The expected costs and QALYs were discounted at 3% per year^14^ for the base-case analysis from age 55 years.

### Resource Use and Costs

Direct costs included those due to health care resource uses for screening, diagnosis, and disease management. We assumed an average of 2 standard biopsies would be undertaken per man diagnosed due to symptoms based on data from the SPBR. Indirect costs regarding productivity losses due to job absenteeism and morbidity were calculated using the human capital method and considering age 65 years as the general age for retirement. Unit costs were extracted from a previous study^18^ and official price lists and converted to the calendar year 2020 using the consumer price index^19^ except for pharmaceuticals, which were recalculated based on the latest data (eTables 1 and 2 in the Supplement).

### Health Outcomes

QALYs were calculated by summing up the products of health state values and the duration in each health state. The health state values alongside screening, diagnosis, and disease management were based on a review by Magnus et al^20^ and a supplement from our previous study^21^ using the disease-specific Patient Oriented Prostate Utility Scale-Utility. These values were multiplied by the background age-specific health state value from the general population in Sweden.^22^ We followed the health state durations from Heijnsdijk et al^10^ except where we assumed 18 and 12 months for metastasis and palliative care, respectively, based on information from the palliative register in Sweden (eTable 3 in the Supplement).^23^

### Statistical Analysis

One-way sensitivity analyses were conducted to assess the following factors: (1) the health state value set reviewed by Heijnsdijk et al^24^; (2) 3-yearly and 5-yearly screening intervals for men aged 55 to 69 years; (3) screening ages between 50 and 74 years according to STHLM3-MRI study^3^; (4) use of MRIs for men with interval cancers during the screening ages and men with cancers detected due to symptoms outside of the screening ages for strategy 3; (5) plus or minus 50% adjustments to biopsy and MRI costs; (6) the substitution of combined biopsies with targeted biopsies alone for strategy 3; (7) biennial rescreening intervals for men with PSA levels of 1 ng/mL or higher and 6-yearly rescreening interval for men with PSA levels below 1 ng/mL; (8) 80% attendance for rescreening, as applied by Getaneh et al^25^; and (9) variation of discount rates between 0% and 5% according to the Swedish guidelines.^14^

Our probabilistic sensitivity analysis considered the joint uncertainties in the test characteristics, costs, and health state values. We assumed that the test characteristics and health state values were logit-normal and all costs were multiplied by a γ variable with shape and scale of 100. The parameter sets were resampled 1000 times. The cost-effectiveness acceptability curve was presented to inform the probability of a strategy being cost-effective relative to other alternatives at a particular cost-effectiveness threshold. This model was implemented in R version 4.0.5 (R Project for Statistical Computing) using microsimulation package version 1.3.7 and the prostata package version 1.3.1 (GitHub). Any tests for statistical significance were 2-sided with *P* < .05.

## Results

### Base Case Analysis

Based on the imputed data, the relative positive fractions comparing the experimental arm using MRI and combined biopsies with using standard biopsies in the standard arm for benign biopsies, cancers from grade groups 1 and 2 or higher were 0.17 (95% CI, 0.14-0.21), 0.28 (95% CI, 0.19-0.40), and 1.06 (95% CI, 0.86-1.30), respectively. Relative to no screening, the screening strategies reduced the prostate cancer related deaths by 6% to 9% over a lifetime period. Compared with using standard biopsy, screening with MRI halved the number of biopsies and reduced the overdiagnosis by 46% across a lifetime.

**Figure 2.**
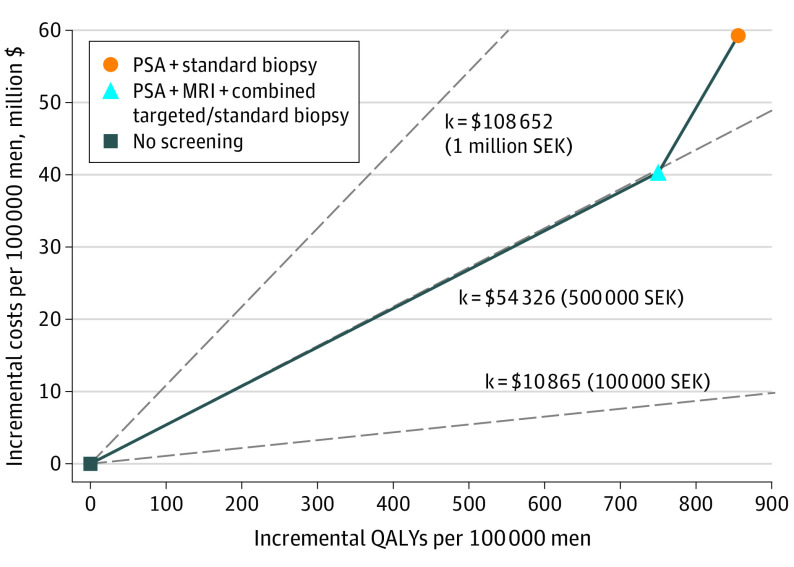
Cost-effectiveness Plane—Base Case, Health Care Perspective, Discounted at 3% ICER indicates incremental cost-effectiveness ratio; MRI, magnetic resonance imaging; PSA, prostate-specific antigen test; QALY, quality-adjusted life-year; SEK, Swedish kronor. Solid lines represent cost-efficiency frontier, dotted lines the thresholds for a low, moderate, high or very high cost per QALY gained in Sweden. The ICER of screening using MRI and combined biopsies relative to no screening was close to the category of a moderate cost per QALY gained. Screening with standard biopsy was classified as a high cost per QALY gained in Sweden.

From a health care perspective and using a 3% discount rate, the ICER comparing no screening with screening using MRI and combined biopsies was $53 736 per QALY gained (strategy 1: cost per 100 000 men, $212.0 million; 1 463 945 QALYs per 100 000 men; strategy 3: cost per 100 000 men, $252.3 million; 1 464 696 QALYs per 100 000 men). This result was classified as a moderate cost per QALY gained in Sweden. Screening with standard biopsies resulted in higher costs (US $271.2 million per 100 000 men vs $252.3 million) and very similar QALY gains (1 464 801 QALYs per 100 00 men vs 1 464 696 QALYs) compared with screening using MRI, with an ICER ($179 856 per QALY gained) classified as very high in Sweden (Table 2; Figure 2; eTables 3-5 in the Supplement).

**Table 2.** Summarized Estimates in Outcomes, Costs, and ICERs—Base Case, Health Care Perspective, 3% Discounted for QALYs and Costs^a^

Strategy	Costs (millions) per 100 000 men, $	QALYs per 100 000 men	ICERs vs strategy 1	ICER strategy 2 vs 3
1. No screening	212.0	1 463 945	NA	NA
3. PSA and MRI with combined targeted and standard biopsies	252.3	1 464 696	53 736	NA
2. PSA and standard biopsy	271.2	1 464 801	69 254	179 856

Abbreviations: ICER, incremental cost-effectiveness ratio; MRI, magnetic resonance imaging; PSA, prostate-specific antigen; QALYs, quality-adjusted life-years.

^a^ The results are presented from age 55 years (conditional on survival to age 55).

### Sensitivity Analyses

**Figure 3.**
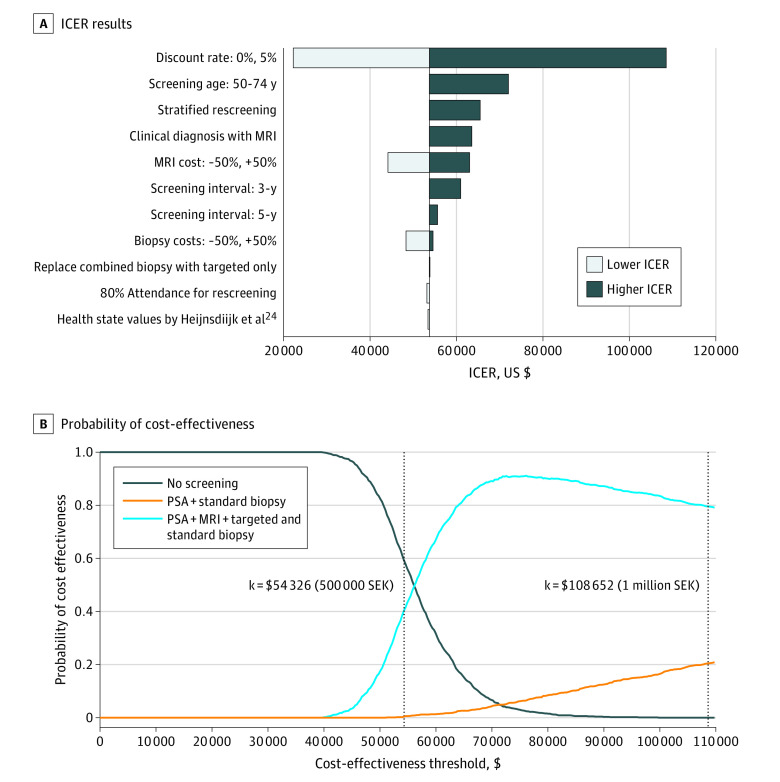
Results From the Sensitivity Analyses ICER indicates incremental cost-effectiveness ratio; MRI, magnetic resonance imaging; PSA, prostate-specific antigen; QALY, quality-adjusted life-year; SEK, Swedish kronor. In panel B, the probabilities of all strategies being cost-effective add up to 100% at a given cost-effectiveness threshold. At a nominal cost-effectiveness threshold of $54 326 (500 000 SEK) per QALY gained, the probability of the screening strategy using PSA and MRI being cost-effective was 40% compared with other strategies. At a nominal cost-effectiveness threshold of $108 652 (1 000 000 SEK) per QALY gained, the probability of screening strategy using PSA and MRI being cost-effectiveness was 85%.

In the 1-way sensitivity analyses (Figure 3A; eTables 6-21 in the Supplement), expanding the screening ages to 50 to 74 years maintained QALYs and increased the costs by 9.1% compared with screening for ages 55 to 69 years with discounting from age 50 years (eTable 9, eTable 21 in the Supplement). Moving to screening for ages 50 to 74 years, the ICER comparing MRI-based screening with no screening increased by 34%. Using PSA-specific rescreening intervals in the MRI pathway resulted in higher costs and QALYs compared with 4-yearly rescreening. Compared with no screening, the MRI pathway with stratified rescreening led to a 21.9% increase in the ICER. For the comparison between 4-yearly screening using MRI and no screening, including MRI for clinical diagnoses outside the screening program increased the ICER by 18.3%. Decreasing and increasing the MRI unit cost by 50% led to a 17.8% decrease and 17.3% increase of the ICERs, respectively. Relative to no screening, a 3-yearly screening increased the ICER by 13.5% while a 5-yearly screening showed small increase of the ICER by 3.5%. Halving the MRI unit cost resulted in a 10% reduction in the ICER. Assuming any of (1) an 80% attendance for the rescreening, (2) an increased biopsy unit cost by 50%, (3) the use of alternative health state values, or (4) the use of targeted biopsy for men with positive MRI results was not associated with a significant change on the ICER. The results were sensitive to the discount rates.

In the probabilistic sensitivity analysis, screening with PSA and MRI was associated with a 40% chance of being cost-effective given a cost-effectiveness threshold of 500 000 SEK (US $54 326) per QALY gained (Figure 3B; eFigure 2 in the Supplement). In contrast, at a cost-effectiveness threshold of 1 000 000 SEK (US $108 652) per QALY gained, this strategy showed an 85% chance of being cost-effective. Screening with PSA and standard biopsy had very low probability of being cost-effective irrespective of the choice of thresholds.

## Discussion

### Main Findings

Compared with no screening, the screening strategies were associated with a reduced rate of prostate cancer–related deaths of 6% to 9% across a lifetime. Screening with PSA followed by MRI and a combined targeted and standard biopsy resulted in an incremental cost-effectiveness ratio that was classified as a moderate cost per QALY gained in Sweden. In relation to traditionally suggested screening with PSA and standard biopsy, screening with MRI reduced the number of both biopsies and overdiagnosis by approximately 50% across a lifetime and had a higher probability of being cost-effective.

### Comparison With Existing Studies and Interpretation of Selected Results

Our primary analysis used a health care perspective, which is different from our previous analyses.^21,26^ The updated Swedish guidelines recommend using a health care perspective rather than a societal perspective, where the latter includes costs due to productivity losses prior to the general retirement age. Individuals who are aged 65 years or over, work part-time, or are unemployed may be discriminated against by this societal perspective, particularly for diseases which have a marked impact at older ages. Using a societal perspective, the ICER comparing screening with MRI with no screening in this study was $61 817 (2020 price), which is higher than our previous projection of $45 628 (2019 price).^26^ The previous study used an adjustment for men with negative MRI results based on data from the Cochrane Review,^21^ whereas the current study used imputed data from the STHLM3-MRI trial. These ICERs also exhibited differences from our model based on the Cochrane review, where the ICER was $100 301.^21^ This variation may largely be explained by the proportion of a negative MRI result being 62% given PSA levels of 3 ng/mL or higher from the STHLM3-MRI screening trial,^7^ which is twice that of the estimation from the Cochrane review (30%) that used data from diagnostic patient cohorts.^5^ Differences were also found in the relative positive fractions of benign biopsies and cancers in grade groups 1 and 2 or higher estimated based on STHLM3-MRI and the Cochrane review, where the former had further reductions in benign biopsies and grade group 1 cancers but detected more cancers in grade group 2 or higher.

To our knowledge, this is the first study assessing the cost-effectiveness of MRI to aid prostate cancer detection in a screening context using test characteristics from a screening-by-invitation trial. Getaneh et al^25^ and Barnett et al^27^ also found that adding MRI to screening was more cost-effective than the classical PSA screening in the Netherlands and US, respectively. However, different clinical and patient characteristics applied in each study: younger screening ages of 50 to 64 years and more frequent screening intervals in the Getaneh et al study,^25^ and a higher PSA threshold of 4 ng/mL and use of a Markov model in the Barnett et al study.^27^ Importantly, only targeted biopsy is referred from positive MRI results by Getaneh et al^25^ and the test parameters were obtained from previous studies^25^ and from different, small studies.^27^ The screening ages used in this study were in line with the ERSPC trial^1^ and Barnett et al.^27^ Screening during ages 50 to 74 years increased the ICER by approximately 34% for the comparison between MRI pathway and no screening. However, the traditional PSA pathway was dominated by the MRI pathway, further suggesting that use of MRI and combined biopsies is preferable in a screening context.

### Strengths and Limitations

One major strength of this study is that we used level 1 randomized evidence for the test characteristics from the population-based STHLM3-MRI trial. Another key strength was that our open-source microsimulation model had been carefully calibrated to Sweden and ERSPC, allowing us to estimate the lifetime effects of screening, diagnostic and treatment progress, together with costs and health effects.

Our study had several limitations. First, the test characteristics were estimated for a per-protocol analysis, rather than an intention-to-treat perspective. Note that the current model incorporated nonparticipation and noncompliance for the screening strategies. Second, the test characteristics from the STHLM3-MRI trial were assumed to remain the same in the subsequent screenings. Data are available from the second screen from the Göteborg 2 study; however, the results from that study were based on younger screening ages (50 to 60 years) and a 2-yearly screening interval.^28^ Relevant data from the subsequent screenings of the STHLM3-MRI Reinvite study^3^ are expected to provide valuable input. Third, although the current results are specific to the Swedish context, the open-source model can be adapted to investigate cost-effectiveness in other populations.

## Conclusions

This economic evaluation based on a microsimulation model found that, given a screening context, the incorporation of MRI with subsequent combined targeted and standard biopsies in quadrennial screening for men aged 55 to 69 years was classified as a moderate cost per QALY gained in Sweden and had a high probability to be more cost-effective relative to the traditional PSA screening pathway. Magnetic resonance imaging was more effective and cost-effective in this population-based study compared with estimates from a recent Cochrane Review based on diagnostic patient cohorts.^5^

## Notes

Supplement. **eFigure 1.** Natural History Simulation Model
eMethods.
**eTable 1.** Substances Used for Prostate Cancer Treatment **eTable 2.** Resources and Unit Costs Characterization **eTable 3.** Health State Values Used in the Cost-effectiveness Analysis **eTable 4.** Summarized Predictions in Outcomes, Costs, and ICERs—Base Case, Healthcare Perspective, 3% Discounted **eTable 5.** Summarized Predictions in Outcomes, Costs, and ICERs—Base Case, Societal Perspective, 3% Discounted **eTable 6.** Summarized Predictions in Outcomes, Costs, and ICERs—Health State Value Set Reviewed by Heijnsdijk et al **eTable 7.** Summarized Predictions in Outcomes, Costs, and ICERs—Triennial Screening **eTable 8.** Summarized Predictions in Outcomes, Costs, and ICERs—5-Yearly Screening **eTable 9.** Summarized Predictions in Outcomes, Costs, and ICERs—Screening Age 50-74 Years **eTable 10.** Summarized Predictions in Outcomes, Costs, and ICERs—Adding MRI to Clinical Diagnosis for Strategy III **eTable 11.** Summarized Predictions in Outcomes, Costs, and ICERs—Reduce Biopsy Cost by 50% **eTable 12.** Summarized Predictions in Outcomes, Costs, and ICERs—Increase Biopsy Cost by 50% **eTable 13.** Summarized Predictions in Outcomes, Costs, and ICERs—Reduce MRI Cost by 50% **eTable 14.** Summarized Predictions in Outcomes, Costs, and ICERs—Increase MRI Cost by 50% **eTable 15.** Summarized Predictions in Outcomes, Costs, and ICERs—Replacing Combined Biopsies by Targeted Biopsy for Strategy III **eTable 16.** Summarized Predictions in Outcomes, Costs, and ICERs—Stratified Rescreening **eTable 17.** Summarized Predictions in Outcomes, Costs, and ICERs—80% attendance of rescreening **eTable 18.** Summarized Predictions in Outcomes, Costs, and ICERs—Healthcare Perspective, Discount Rate 0% **eTable 19.** Summarized Predictions in Outcomes, Costs, and ICERs—Healthcare Perspective, Costs, and QALYs Discounted at 5% **eTable 20.** Summarized Predictions in Outcomes, Costs, and ICERs—Healthcare Perspective, Costs Discounted at 3%, QALYs Undiscounted **eTable 21.** Summarized predictions in Outcomes, Costs, and ICERs—Base Case, Discounted From Age 50 Years **eFigure 2.** Cost-effectiveness Plane of the Probabilistic Sensitivity Analysis
