# Supplementary material for: Cost-effectiveness of Prostate Cancer Screening Using Magnetic Resonance Imaging or Standard Biopsy Based on the STHLM3-MRI Study
Source: JAMA Oncol. 2022 Nov 10;9(1):88–94. doi: 10.1001/jamaoncol.2022.5252 (PMC9650623; doi:10.1001/jamaoncol.2022.5252)
Supplement: Supplement. — eFigure 1. Natural History Simulation Model eMethods. eTable 1. Substances Used for Prostate Cancer Treatment eTable 2. Resources and Unit Costs Characterization eTable 3. Health State Values Used in the Cost-effectiveness Analysis eTable 4. Summarized Predictions in Outcomes, Costs, and ICERs—Base Case, Healthcare Perspective, 3% Discounted eTable 5. Summarized Predictions in Outcomes, Costs, and ICERs—Base Case, Societal Perspective, 3% Discounted eTable 6. Summarized Predictions in Outcomes, Costs, and ICERs—Health State Value Set Reviewed by Heijnsdijk et al eTable 7. Summarized Predictions in Outcomes, Costs, and ICERs—Triennial Screening eTable 8. Summarized Predictions in Outcomes, Costs, and ICERs—5-Yearly Screening eTable 9. Summarized Predictions in Outcomes, Costs, and ICERs—Screening Age 50-74 Years eTable 10. Summarized Predictions in Outcomes, Costs, and ICERs—Adding MRI to Clinical Diagnosis for Strategy III eTable 11. Summarized Predictions in Outcomes, Costs, and ICERs—Reduce Biopsy Cost by 50% eTable 12. Summarized Predictions in Outcomes, Costs, and ICERs—Increase Biopsy Cost by 50% eTable 13. Summarized Predictions in Outcomes, Costs, and ICERs—Reduce MRI Cost by 50% eTable 14. Summarized Predictions in Outcomes, Costs, and ICERs—Increase MRI Cost by 50% eTable 15. Summarized Predictions in Outcomes, Costs, and ICERs—Replacing Combined Biopsies by Targeted Biopsy for Strategy III eTable 16. Summarized Predictions in Outcomes, Costs, and ICERs—Stratified Rescreening eTable 17. Summarized Predictions in Outcomes, Costs, and ICERs—80% attendance of rescreening eTable 18. Summarized Predictions in Outcomes, Costs, and ICERs—Healthcare Perspective, Discount Rate 0% eTable 19. Summarized Predictions in Outcomes, Costs, and ICERs—Healthcare Perspective, Costs, and QALYs Discounted at 5% eTable 20. Summarized Predictions in Outcomes, Costs, and ICERs—Healthcare Perspective, Costs Discounted at 3%, QALYs Undiscounted eTable 21. Summarized predictions in O [file jamaoncol-e225252-s001.pdf]

Supplemental Online Content

Hao S, Discacciati A, Eklund M, et al. Cost-effectiveness of prostate cancer screening using magnetic resonance imaging or standard biopsy based on the STHULM3-MRI study. *JAMA Oncol.* Published online November 10, 2022.
doi:10.1001/jamaoncol.2022.5252

eFigure 1. Natural History Simulation Model
eMethods.
eTable 1. Substances Used for Prostate Cancer Treatment
eTable 2. Resources and Unit Costs Characterization
eTable 3. Health State Values Used in the Cost-effectiveness Analysis
eTable 4. Summarized Predictions in Outcomes, Costs, and ICERs—Base Case, Healthcare Perspective, 3% Discounted
eTable 5. Summarized Predictions in Outcomes, Costs, and ICERs—Base Case, Societal Perspective, 3% Discounted
eTable 6. Summarized Predictions in Outcomes, Costs, and ICERs—Health State Value Set Reviewed by Heijnsdijk et al
eTable 7. Summarized Predictions in Outcomes, Costs, and ICERs—Triennial Screening
eTable 8. Summarized Predictions in Outcomes, Costs, and ICERs—5-Yearly Screening
eTable 9. Summarized Predictions in Outcomes, Costs, and ICERs—Screening Age 50-74 Years
eTable 10. Summarized Predictions in Outcomes, Costs, and ICERs—Adding MRI to Clinical Diagnosis for Strategy III
eTable 11. Summarized Predictions in Outcomes, Costs, and ICERs—Reduce Biopsy Cost by 50%
eTable 12. Summarized Predictions in Outcomes, Costs, and ICERs—Increase Biopsy Cost by 50%
eTable 13. Summarized Predictions in Outcomes, Costs, and ICERs—Reduce MRI Cost by 50%
eTable 14. Summarized Predictions in Outcomes, Costs, and ICERs—Increase MRI Cost by 50%
eTable 15. Summarized Predictions in Outcomes, Costs, and ICERs—Replacing Combined Biopsies by Targeted Biopsy for Strategy III
eTable 16. Summarized Predictions in Outcomes, Costs, and ICERs—Stratified Rescreening
eTable 17. Summarized Predictions in Outcomes, Costs, and ICERs—80% attendance of rescreening
eTable 18. Summarized Predictions in Outcomes, Costs, and ICERs—Healthcare Perspective, Discount Rate 0%
eTable 19. Summarized Predictions in Outcomes, Costs, and ICERs—Healthcare Perspective, Costs, and QALYs Discounted at 5%
eTable 20. Summarized Predictions in Outcomes, Costs, and ICERs—Healthcare Perspective, Costs Discounted at 3%, QALYs Undiscounted

© 2022 Hao S et al. *JAMA Oncology.*

eTable 21. Summarized predictions in Outcomes, Costs, and ICERs—Base Case, Discounted From Age 50 Years
eFigure 2. Cost-effectiveness Plane of the Probabilistic Sensitivity Analysis

This supplemental material has been provided by the authors to give readers additional information about their work.

© 2022 Hao S et al. *JAMA Oncology.*

eFigure 1. Natural history simulation model

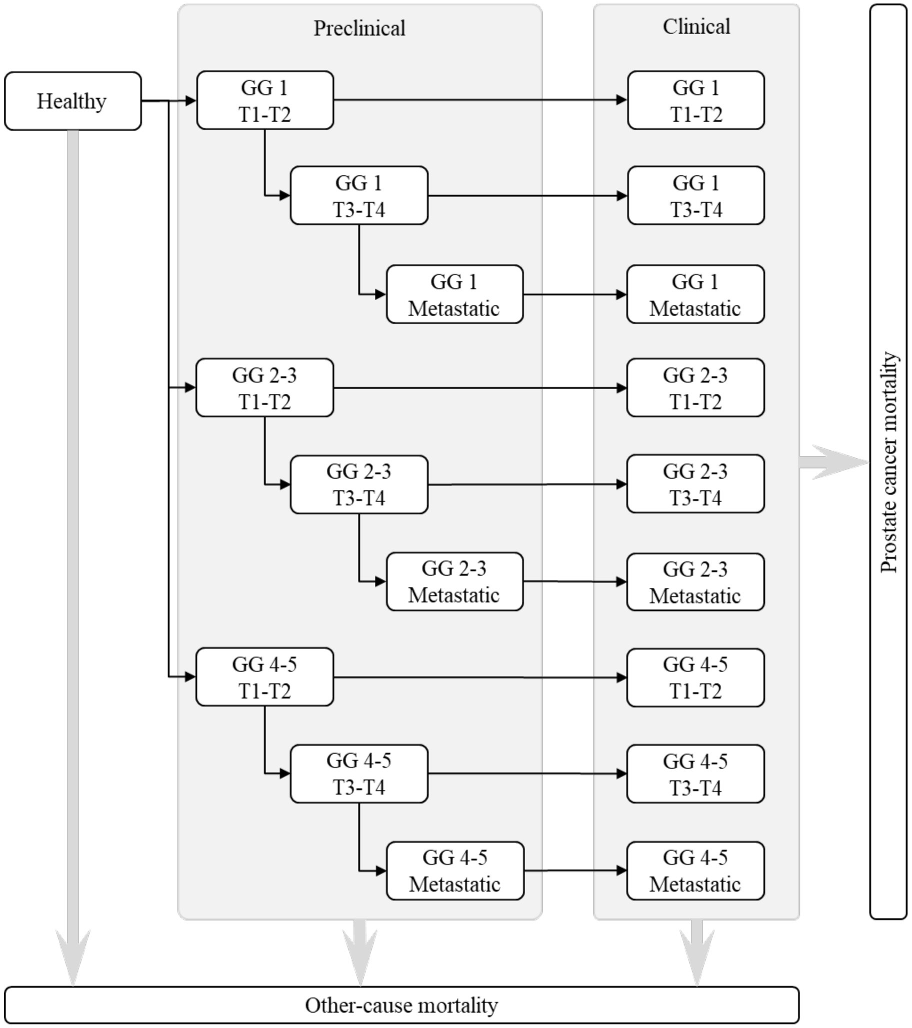

The model reflects cancer onset by ISUP (GG 1; GG 2-3; GG 4-5), with progression by T-stage and to metastatic cancer. Preclinical cancers may be clinically diagnosed, with cause-specific survival from the time of clinical diagnosis. Death due to other causes is represented as a competing event. ISUP: International Society of Urological Pathology: GG: grade group.

© 2022 Hao S et al. *JAMA Oncology.*

eMethods.

Test characteristics parameter adjustment

A1 Model-based imputation (MBI)

Methods

Overall, 276 out of 1532 randomised men with PSA≥3 ng/ml (18%) did not follow the protocol. Specifically, 165 out of 603 men randomised to the standard arm (27%) did not undergo standard biopsy, while 111 out of 929 men randomised to the experimental arm (12%) did not undergo MRI (n=83) or any biopsy even if MRI positive (n=28). Besides MRI and biopsy variables, missing values were observed in the following variables collected at baseline and used in the imputation procedure (see next paragraph): Stockholm3 test score (n=1), family history of prostate cancer (n=204), and previous biopsy procedure (n=56).

We used multivariate imputation by chained equations (MICE) to impute 500 complete datasets. Imputations were performed separately by randomisation arm. The variables included in the MICE procedure were the following: age, PSA, Stockholm3 test score, family history of prostate cancer (yes/no), previous biopsy procedure (yes/no), MRI result [PI-RADS≤2 (negative) or PI-RADS≥3 (positive)], and biopsy result [benign biopsy, Gleason Grade 1 (clinically insignificant) cancer, or Gleason Grade ≥2 (clinically significant cancer)]. Stockholm 3 score test was imputed using predictive mean matching, with binary variables modelled using logistic regression, while the biopsy result was modelled using a polytomous (multinomial) logistic regression. In the experimental arm, the imputed biopsy results for those men with a negative MRI result were not further used.

The MICE procedure was performed using R version 3.6.3 and the package mice v 3.9.0. [1]

For the estimation of the means and confidence intervals for the relative positive fraction, let n_1 and n_2 be the number of individuals with PSA≥3ng/mL in the experimental and control arms, respectively, and let p_{1k} and p_{2k} be the imputed proportion of those individuals with PSA≥3ng/mL who had a biopsy of a particular type k (e.g. negative biopsy, GG=1 or GG>=2) in the experimental and control arms, respectively. The relative positive fraction for a particular type was calculated by $RPF_k=p_{1k}/p_{2k}$. The variance for the relative positive fraction for a particular type for a given imputed sample was calculated using the approximation that $var(log(RPF_k)) \sim= (1/p_{1k}-1)/n_1 + (1/p_{2k}-1)/n_2$. The mean and variance for the relative positive fractions on the log scale were calculated across the imputation sets using Rubin's rule. The confidence intervals for the relative positive fractions were calculated on the log scale.

A2 Adjustment for the disease grading of participants with negative MRI results in the experimental arm [2]

First we tabulated the number of cases from the standard arm by ISUP grading, either benign biopsies, GG=1 or GG≥2 cancers. In total, there were 603 cases with PSA≥3ng/mL, in which 165 noncompliant cases to the protocol required MBI.

Table SA1

Before MBI – Standard arm	PSA<3ng/mL	PSA≥3ng/mL	Total	PSA≥3ng/mL GG (%)
N/A	79	**165**	244	
Benign biopsies (GG=0)	146	259	405	59.1%
GG=1 cancers	42	73	115	16.7%
GG≥2 cancers	51	106	157	24.2%
Total	318	603	921	

*165 for MBI

© 2022 Hao S et al. *JAMA Oncology.*

Results after MBI were listed below in Table SA2. The proportion of cases by ISUP grading after MBI was similar to the proportions observed before MBI.

Table SA2

After MBI – Standard arm	PSA<3ng/mL	PSA≥3ng/mL	Total	PSA≥3ng/mL GG (%)
N/A				
Benign biopsies (GG=0)		368*		61.0%
GG=1 cancers		101*		16.7%
GG≥2 cancers		134*		22.2%
Total		603		

*The imputed value 368, 101 and 134 were denoted as r1, r2 and r3

For the number of cases from the experimental arm, 929 cases with PSA≥3ng/mL were tabulated by ISUP grading, in which 83 cases noncompliant to the protocol that should have had MRI and 28 cases with positive MRI results but did not undertake biopsies were considered for MBI.

Table SA3

Before MBI – Experimental arm	PSA<3ng/mL	PSA≥3ng/mL			Total	MRI+ GG (%)
		MRI NA	MRI-	MRI+ TBx/SBx		
N/A	318	78	485	**28***	909	
Benign biopsies (GG=0)	50	2	24	79	155	*26,6%*
GG=1 cancers	20	0	6	35	61	*11,8%*
GG≥2 cancers	55	3	6	183	247	*61,6%*
Total	443	**83***	521	325	1372	
			61.6%	*38.4%*		

*28 and 83 for MBI

Results after MBI for the experimental arm were listed below. We also calculated the relative positive fractions (rPFs) comparing the proportions of number of cases by ISUP grading after MBI (GG (%)) between the experimental arm and standard arm.

Table SA4

After MBI – Experimental arm	PSA<3ng/mL	PSA≥3ng/mL		Total	MRI+ GG (%)	GG (%)
		MRI-	MRI+ TBx/SBx			
N/A	318			318		
Benign biopsies (GG=0)	50		98	148	*27.2%*	*10.5%*

© 2022 Hao S et al. *JAMA Oncology.*

GG=1 cancers	20		43	63	*11.9%*	*4.6%*
GG≥2 cancers	55		219	274	*60.8%*	*23.6%*
Total	443	569	360	1372		
		61.2%	*38.8%*			

*Relative positive fractions comparing GG (%) from the experimental arm after MBI with GG (%) from the standard arm after MBI

Based on the per-protocol data observed from the experimental arm in the STHLM3-MRI trial excluding any cases with S3M≥25%, the number of cases using MRI+TBx/SBx by ISUP grading were tabulated in the table below. We also tabulated the number of cases assuming using MRI and SBx to detect cancers. Note that here assuming MRI+SBx was only for the estimation of test characteristics. In clinical practice, patients with positive MRI results were further examined with either TBx or combined TBx/SBx. The rPFs were calculated comparing the number of cases by ISUP grading between MRI+TBx/SBx and the assumed MRI+SBx.

Table SA5

Observed – Experimental arm	MRI+TBx/SBx	MRI+SBx	**rPFs**
Benign biopsies (GG=0)	77	105	0.733
GG=1 cancers	35	52	0.673
GG≥2 cancers	177	132	1.341
Total	289	289	

We then applied the rPFs from the step above given the imputed data of MRI+TBx/SBx, to extrapolate the number of cases assuming using MRI+SBx for the imputed data. The proportion of cases by ISUP grading for the extrapolated data were calculated accordingly.

Table SA6

After MBI – Experimental arm	MRI+TBx/SBx	MRI+SBx	MRI+SBx GG (%)
Benign biopsies (GG=0)	98	134	37.0
GG=1 cancers	43	64	17.7
GG≥2 cancers	219	163	45.3
Total	360	361	

*The extrapolated value 134, 64 and 1363 were denoted as q1, q2 and q3

Given the total number of cases (603) in the standard arm after MBI from Table SA2 and the ratio between MRI- and MRI+ from Table SA4 (569:360), assuming MRI was used in the standard arm, the extrapolated total number of cases for MRI- and MRI+ were 369 and 234. Applying the proportion of cases by ISUP grading calculated from Table SA6, the number of cases for MRI+SBx was tabulated as 87, 41 and 106 for benign biopsies, GG=1 and GG≥2 cancers, respectively. The number of cases by ISUP grading for MRI- was calculated by deducting the number of cases by ISUP grading of MRI+SBx from the number of cases by ISUP grading of total SBx.

Table SA7

© 2022 Hao S et al. *JAMA Oncology.*

After MBI – Standard arm	MRI-	MRI+SBx	Total SBx	MRI- GG (%)
Benign biopsies (GG=0)	281	87	368	76.2
GG=1 cancers	60	41	101	16.1
GG≥2 cancers	28	106	134	7.6
Total	369	234	603	

Given the data from Table SA4 and the MRI- GG (%) calculated from Table SA7, the number of cases by ISUP grading for MRI- was extrapolated in Table SA8 for the experimental arm after MBI.

Table SA8

After MBI – Experimental arm	MRI-	MRI+TBx/SBx	Total
Benign biopsies (GG=0)	434	98	532
GG=1 cancers	92	43	135
GG≥2 cancers	43	219	262
Total	569	360	929

*The extrapolated value 434, 92 and 43 were denoted as x, y and z

The matrix below showed the observed data from the experimental arm before MBI. The data in the coloured area were tabulated based on the full data availability of both SBx and TBx.

Matrix S1 Cases by type of test and ISUP grading before MBI

	MRI- & SBx	MRI + TBx			Combined	**MRI+SBx**
SBx		GG=0	GG=1	GG≥2	Total	Total
GG=0 (benign biopsies)		77	7	21		105
GG=1		19	9	24		52
GG≥2		11	7	114		132
	521	107	23	159	810	289
**Total**	521	289				
	Given	Benign biopsies (GG=0)			77	
		GG=1			35	
		GG≥2			177	

After MBI, given the extrapolated data from Table SA8, we first filled in the left side of the matrix below. From Matrix S1, the proportion of the cases of a specific combination of ISUP grading from SBx and MRI+TBx out of the total number of GG=0, GG=1 and GG≥2 cancers were calculated. For instance, the proportion of

© 2022 Hao S et al. *JAMA Oncology.*

cases having GG=1 by SBx and GG=1 by MRI+TBx out of total GG=1 cases was calculated as 9/35=0.257. We applied the proportion of cases in each cell out of the corresponding total number of cases under the same ISUP grading from Matrix S1 to Matrix S2.

Matrix S2: Cases by type of test and ISUP grading after MBI

	MRI- & SBx	MRI + TBx			Combined	**SBx**	
SBx		GG=0	GG=1	GG≥2	Total	Total	
GG=0 (benign biopsies)	434	98	9	26	532	566	
GG=1	92	23	11	30	135	156	
GG≥2	43	14	9	141	262	207	
	569	135	28	197	929	929	
**Total**	569	360					
	Given	Benign biopsies (GG=0)			98		
		GG=1			43		
		GG≥2			219		

Please note that due to rounding, the added values of the first three rows in the matrix above may not be exactly the same as the values showed in the fourth row.

The test characteristics calculated based on Matrix S2:

**Test characteristics**			
**Test characteristics**		**Value**	**95% CI**
Probability of positive MRI results (GG=0)	Pr (MRI+ | GG*=0)	0.184	
Probability of positive MRI results (GG=1)	Pr (MRI+ | GG*=1)	0.319	
Probability of positive MRI results (GG≥2)	Pr (MRI+ | GG*≥2)	0.835	
False negative rate of SBx strategy (GG=1)	Pr (SBx- | GG*=1)	0.064	
False negative rate of SBx strategy (GG≥2)	Pr (SBx- | GG*≥2)	0.099	

CI: Confidence Interval; GG: Internal Society of Pathological Urology Grade Group; MRI: Magnetic Resonance Imaging; SBx: standard biopsy.

The test characteristics using the procedures described after MBI:

**Test characteristics**			
**Test characteristics**		**Value**	**95% CI**
Probability of positive MRI results (GG=0)	Pr (MRI+ | GG*=0)	0.184	(0.147, 0.229)
Probability of positive MRI results (GG=1)	Pr (MRI+ | GG*=1)	0.317	(0.198, 0.465)
Probability of positive MRI results (GG≥2)	Pr (MRI+ | GG*≥2)	0.837	(0.643, 0.936)
False negative rate of SBx strategy (GG=1)	Pr (SBx- | GG*=1)	0.063	(0.028, 0.135)

© 2022 Hao S et al. *JAMA Oncology.*

False negative rate of SBx strategy (GG≥2)	Pr (SBx- | GG*≥2)	0.099	(0.064, 0.151)

CI: Confidence Interval; GG: Internal Society of Pathological Urology Grade Group; MRI: Magnetic Resonance Imaging; SBx: standard biopsy.

A3 Comparison of relative positive fractions in detecting benign biopsies, GG=1 and GG≥2 cancers using MRI with combined targeted and standard biopsies relative to using standard biopsies alone

	Estimates based on the STHLM3-MRI study	95% CI	Estimates based on selected studies from the Cochrane review	95% CI	Chi-square test
Benign biopsies	0.17	(0.14, 0.21)	0.37	(0.28, 0.48)	<0.000
GG=1 cancers	0.27	(0.19, 0.40)	0.66	(0.57, 0.76)	<0.000
GG≥2 cancers	1.06	(0.86, 1.30)	0.64	(0.49, 0.85)	<0.004

CI: Confidence Interval; GG: Internal Society of Pathological Urology Grade Group; MRI: Magnetic Resonance Imaging.

Resource characterisation, costs characterisation and health state value sets

The input data for resources use and unit costs of used resources were extracted from multiple price lists of case in Stockholm Region, a report from the National Board of Health and Welfare (NBHW) and our previous study [3-11]. The duration of the lost production during to diagnosis, treatment, post treatment recovery and terminal care were collected from Heijnsdijk et al [12]. For long-term sick leave by patients with metastatic prostate cancer or patients under palliative therapy, the proportion under employment was 7.68% and the average days of sick leave was approximately 68 days in 2016 [11]. The employment ratio for the general population in Sweden were 0.884, 0.778, 0.186 and 0 for age groups 45-54, 55-64, 65-74 and over 74 years, respectively in 2020 [13]. The average monthly salary of the general population in Sweden was €3,442 in 2020 [14], with an additional 37.20% for employer and social contributions [15]. All costs in Swedish kroner were first aligned to the calendar year 2020 by using the consumer price index [16] and then converted to United States Dollar (USD, $) 2020 price by the exchange rate (1 USD = 9.2037 SEK) from the national bank [17] .

Regarding the average pharmaceutical costs per patient due to prostate cancer in Sweden, we considered prescribed drugs (förmånsläkedemel/Recept) and requisition drugs (rekvisition-läkedemel) in this study. A list with drugs used in Sweden for treating prostate cancer has been made by referring the information from the Pharmaceutical Specialties in Sweden [18] and the drug list used for prostate cancer from Internetmedicin in Sweden [19]. It included 17 substances for treating prostate cancer. We requested the data of drug costs at a substance level (ATC5) for the usage of all patients in Sweden in year 2020 from The Concise database of the Swedish eHealth Agency (E-Hälsomyndigheten) [20], which is the source that can provide both the costs of prescribed and requisition drugs. As most of the drugs in the list have multiple indications, the costs of each substance dedicated for treating prostate cancer were estimated. For those substances used previously, we applied the proportion of drug usage estimated for prostate cancer from a previous study [11]. Three substances were launched after the year 2016 for prostate cancer treatment and one substance was approved with a new indication of prostate cancer. However, for two of the newly launched substances, the substance with a new indication for prostate cancer and one previously listed substance, did not generate any costs in the year 2020. In addition, one substance was not available from 2019 and one substance was available. In total, 12 substances were used for the estimation. Furthermore, we mapped each substance with the risk level of eligible patients, either localised – intermediate risk, localised – high risk, locally advanced, regionally metastatic or distant metastasis. This risk level was aligned with the

© 2022 Hao S et al. *JAMA Oncology.*

National Prostate Cancer Register (NPCR), from which we retrieved the number of patients by risk level in the year 2020 in Sweden [21]. Due to data restrictions, we estimated the average yearly drug costs per patient by aggregating the costs for each substance used for prostate cancer and divided by the number of patients registered in the National Prostate Cancer Register (NPCR) in Sweden [21]. For the substance list, see Table S1.

© 2022 Hao S et al. *JAMA Oncology.*

Table S1 Substances used for prostate cancer treatment (2020)

ATC5	Substance	Administration	Category	Type	Costs in 2020 for PCa	Use for PCa as prescribed drug*	Use for PCa as requisition drug*
L01CD02	Docetaxel	Intraveneous infusion	Chemo therapy	Cytostatics	Yes	7.0%	28.6%
L01CD04	Cabazitaxel	Intraveneous infusion	Chemo therapy	Cytostatics	Yes	-	100%
L01DB07	Mitoxantrone	Intravenous infusion	Chemo therapy	Anthracenedione antineoplastic agent	Yes	-	100%
L01XK01	Olaparib	Oral	Antineoplastic drugs	PARP inhibitors	No	-	-
L02AA02	Polyestradiol phosphate	Intramuscular injection	Hormone therapy	Estrogen	No	-	-
L02AE01	Buserelin	Subcutaneous injection	Hormone therapy	GnRH analogue	Yes	29.2%	29.2%
L02AE02	Leuprorelin	Intramuscular injection or Subcutaneous injection	Hormone therapy	GnRH analogue	Yes	95.2%	100%
L02AE03	Goserelin	Subcutaneous injection	Hormone therapy	GnRH analogue	Yes	65.6%	33.2%
L02AE04	Triptorelin	Subcutaneous injection	Hormone therapy	GnRH analogue	Yes	46.7%	35.1%
L02BB03	Bicalutamide	Oral	Hormone therapy	Antiandrogen	Yes	99.8%	100%
L02BB04	Enzalutamide	Oral	Novel hormone therapy	Antiandrogen	Yes	100%	100%
L02BB05	Apalutamide	Oral	Novel hormone therapy	Antiandrogen	No	-	-
L02BB06	Darolutamide	Oral	Novel hormone therapy	Antiandrogen	Yes	100%	-
L02BX02	Degarelix	Subcutaneous injection	Hormone therapy	GnRH antagonist	Yes	100%	100%
L02BX03	Abiraterone	Oral	Novel hormone therapy	Androgen synthesis inhibitors	Yes	100%	100%
V10BX02	Samarium-153	Intraveneous infusion	Radionuclide treatment		No	-	-
V10XX03	Radium-223	Intraveneous infusion	Radionuclide treatment		No	-	-

Source: Pharmaceutical Specialties in Sweden (FASS)[18]; Internetmedicin in Sweden[19]; Swedish eHealth Agency (E-Hälsomyndigheten)[20]; *Hao et al (2020)[11]
GnRH: Gonadotropin-releasing hormone; PARP: Poly (ADP-ribose) polymerase; PCa: Prostate cancer.

© 2022 Hao S et al. *JAMA Oncology.*

Table S2 Resources and unit costs characterization (2020 price)

Module/Procedure	Unit cost ($)	Resource use	Costs ($)	Source	Lost production	Unit	Years of lost production	Source
**Diagnosis**								
PSA test at primary care								
GP visit	166	0.2	33	[3-7]	2	Hour	2/24/365.25	[12]
PSA test analysis	6	1	6	[8]				
**Total costs**			**40**		**2**	**Hour**	**2/24/365.25**	
Biopsy at outpatient care: standard biopsy								
Specialist and nurse consultation	159	1	159	[9]	2	Hour	2/24/365.25	[12]
Standard biopsy	327	1	327	[9]				
Pathology	471	1	471	[8]		-	-	
Nurse consultation	43	1	43	[9]	-	-	-	
**Total costs**			**1 000**		**4**	**Hour**	**4/24/365.25**	
Biopsy at outpatient care: MRI and combined biopsies								
Specialist and nurse consultation	159	1	159	[9]	2	Hour	2/24/365.25	[12]
Targeted and standard biopsies	491	1	491	[9]				
MRI	380	1	380	[9]	2	Hour	2/24/365.25	Expert opinion
Pathology	471	1	471	[8]		-	-	
Nurse consultation	43	1	43	[9]	-	-	-	
**Total costs**			**1 544**		**4**	**Hour**	**4/24/365.25**	
Biopsy at outpatient care: MRI and targeted biopsies								
Specialist and nurse consultation	159	1	159	[9]	2	Hour	2/24/365.25	[12]
Targeted and standard biopsies	325	1	325	[9]				
MRI	380	1	380	[9]	2	Hour	2/24/365.25	Expert opinion
Pathology	471	1	471	[8]		-	-	
Nurse consultation	43	1	43	[9]	-	-	-	
**Total costs**			**1 378**		**4**	**Hour**	**4/24/365.25**	
**Treatment**								
Active surveillance: standard biopsy								
Specialist and nurse consultation	159	1	159		2	Hour	2/24/365.25	[12]
PSA test sampling	40	3	119	[7]	2*3	Hour	2*3/24/365.25	[12]
PSA test analysis	6	3	19	[8]				
Standard biopsy	327	0.33	108	[9]	2*0.33	Hour	2*0.33/24/365.25	[12]

© 2022 Hao S et al. *JAMA Oncology.*

Pathology	471	0.33	155	[8]		-	-	
**Total cost *(Annual cost)***			**560**		9.32	Hour	**9.32/24/365.25**	
Active surveillance: MRI and combined biopsies								
Specialist and nurse consultation	159	1	159	[9]	2	Hour	2/24/365.25	[12]
PSA test sampling	40	3	119	[7]	2*3	Hour	2*3/24/365.25	[12]
PSA test analysis	6	3	19	[8]				
MRI	380	0.33	125	[9]	2*0.33	Hour	2*0.33/24/365.25	Expert opinion
Standard and targeted biopsies	491	0.33	162	[9]	2*0.33	Hour	2*0.33/24/365.25	[12]
Pathology	471	0.33	155	[9]		-	-	
**Total cost *(Annual cost)***			**739**		9.32	Hour	**9.32/24/365.25**	
Active surveillance: MRI and targeted biopsy								
Specialist and nurse consultation	159	1	159	[9]	2	Hour	2/24/365.25	[12]
PSA test sampling	40	3	119	[7]	2*3	Hour	2*3/24/365.25	[12]
PSA test analysis	6	3	19	[8]				
MRI	380	0.33	125	[9]	2*0.33	Hour	2*0.33/24/365.25	Expert opinion
Targeted biopsy	325	0.33	107	[9]	2*0.33	Hour	2*0.33/24/365.25	[12]
Pathology	471	0.33	155	[9]		-	-	
**Total cost *(Annual cost)***			**685**		9.32	Hour	**9.32/24/365.25**	
Radical prostatectomy: robot-assistant								
Robot assisted surgery	12 822	1	12 822	[10]	6	Week	6/52	[12]
Specialist and nurse consultation	159	1	159	[9]				
RT	14 009	0.25	3 502	[10]				
**Total cost**			**16 483**		**6**	**Week**	**6/52**	
Radiation therapy								
Oncologist consultation - new visit	434	1	434	[6]	8	Week	8/52	[12]
Oncologist consultation - further visit	187	1	187	[6]				
Nurse visit	43	20	869	[9]				
RT	700	20	14 009	[10]				
Hormone therapy-yearly	7 501	0.2	1 500	[11]				
**Total cost**			**16 999**		**8**	**Week**	**8/52**	
Metastatic								[11]
Yearly drug cost/patient	15 778	1	15 778	[11]	67.52	Day	67.52/365.25	
**Total cost *(Annual cost)***			**15 778**		**67.52**	**Day**	**67.52/365.25**	
**Post treatment follow-up**								

© 2022 Hao S et al. *JAMA Oncology.*

Post treatment follow-up: first year								
Specialist and nurse consultation - Physical	159	1	159	[9]	2	Hour	2/24/365.25	[12]
PSA test sampling	40	1	40	[7]				
PSA test analysis	6	1	6	[8]				
**Total cost *(Annual cost)***			**205**		**2**	**Hour**	**2/24/365.25**	
Post treatment follow-up: years following								
Specialist consultation - Tele follow-up	16	1	16	[9]	2	Hour	2/24/365.25	[12]
PSA test sampling	40	1	40	[7]				
PSA test analysis	6	1	6	[8]				
**Total cost *(Annual cost)***			**62**		**2**	**Hour**	**2/24/365.25**	
**Palliative therapy**								
Yearly drug cost/patient	17 959	1	17 959	[20,21]	67.52	Day	67.52/365.25	[11]
**Total cost *(Annual cost)***			**17 959**		**67.52**	**Day**	**67.52/365.25**	
**Terminal illness**								
Yearly drug cost/patient	8 980	1	8 980	[11]	6	Month	6/12	[12]
**Total cost**			**8 980**		**6**	**Month**	**6/12**	

GP: General Practitioner; MRI: Magnetic Resonance Imaging; PSA: Prostate-specific Antigen; RT: Radiation Therapy

Table S3 Health state values used in the cost-effectiveness analysis

**Health state**	**PORPUS-U**	**95% CI**	**Duration**	**Unit**	**Background Health State Values**	
PSA test	0.99	(0.99-1.00)	1	Week	**Age (years)**	**Base case**
Biopsy (TBx/SBx; SBx)	0.90	(0.87-0.94)	3	Week	20-29	0.91
Cancer diagnosis	0.80	(0.75-0.85)	1	Month	30-39	0.90
Active surveillance	0.98	(0.97-0.99)	7	Year	40-44	0.86
Radical prostatectomy (part1)	0.86	(0.76-0.96)	2	Month	45-49	0.86
Radical prostatectomy (part2)	0.90	(0.84-0.97)	10	Month	50-54	0.84
Radiation therapy (part1)	0.89	(0.87-0.91)	2	Month	55-59	0.84
Radiation therapy (part2)	0.92	(0.90-0.94)	10	Month	60-64	0.83
Metastatic	0.80	Not reported	18	Month	65-69	0.83
Post recovery period	0.93	(0.91-0.95)	9	Year	70-74	0.81
Palliative therapy	0.68	(0.64-0.71)	12	Month	75-79	0.81
Terminal illness	0.40	Not reported	6	Month	80+	0.74

© 2022 Hao S et al. *JAMA Oncology.*

Source: PORPUS-U value set: Hao et al [22] and Magnus et al [23]; Duration of health states: Heijnsdijk et al [12], Swedish palliative register [24]; Background health state values: Burström et al [25]. PORPUS-U: The Patient Oriented Prostate Utility Scale-Utility; PSA: Prostate-Specific Antigen; SBx: systematic biopsy; TBx/SBx: combined targeted and systematic biopsy

© 2022 Hao S et al. *JAMA Oncology.*

Appendix C. Results of base case analysis and results of sensitivity analysis

Table S4 Summarised predictions in outcomes, costs and ICERs – Base case, healthcare perspective, 3% discounted

**Part A. Lifetime predictions in outcomes and costs for all strategies**			
**Lifetime predictions**	**Strategy I No screening**	**Strategy II PSA and standard biopsy**	**Strategy III PSA, MRI with targeted and standard biopsy**
**Outcomes per 100,000 men**			
Screening tests (age 55-69)	0	283 325	269 524
MRI	0	0	34 846
Biopsy	31 047	72 451	35 714
Diagnosed prostate cancer	16 542	17 401	17 003
Diagnosed prostate cancer – GG≥2	11 908	12 244	12 176
Diagnosed prostate cancer (age 55-69)	2 708	5 628	4 456
Diagnosed prostate cancer (age 55-69) – GG≥2	1 627	3 035	2 762
Over-diagnosed prostate cancer	0	858	461
Prostate cancer deaths	5 799	5 244	5 445
Life years, undiscounted	2 732 833	2 738 081	2 736 400
QALYs, undiscounted	2 186 593	2 189 487	2 188 747
QALYS, discounted at 3%	1 463 945	1 464 801	1 464 696
QALYS, discounted at 5%	1 172 350	1 172 624	1 172 682
**Costs (Million $) per 100,000 men**			
Health care perspective, undiscounted	431.6	493.8	479.5
Health care perspective, discounted at 3%	212.0	271.2	252.3
Health care perspective, discounted at 5%	138.4	193.4	174.5
Societal perspective, undiscounted	442.7	515.3	497.7
Societal perspective, discounted at 3%	220.1	288.2	266.5
Societal perspective, discounted at 5%	145.2	208.1	186.7

**Part B. Incremental cost-effectiveness ratios (ICERs) – Healthcare perspective, 3% discounted for QALYs and costs**				
**Strategy**	**Costs (Million $) per 100,000 men**	**QALYs per 100,000 men**	**ICERs vs. Strategy I**	**ICER Strategy II vs. III**
I No screening	212.0	1 463 945	-	-
III PSA and MRI with combined targeted and standard biopsies	252.3	1 464 696	53 736	-
II PSA and standard biopsy	271.2	1 464 801	69 254	179 856

Note: the results are presented from age 55 years (conditional on survival to age 55). GG: International Society of Urological Pathology Grade Group; ICER: Incremental Cost-Effectiveness Ratio; MRI: Magnetic Resonance Imaging; PSA: Prostate-Specific Antigen; QALY: Quality-Adjusted Life-Year

Table S5 Summarised predictions in outcomes, costs and ICERs – Base case, societal perspective, 3% discounted

**Part A. Lifetime predictions in outcomes and costs for all strategies**			
**Lifetime predictions**	**Strategy I No screening**	**Strategy II PSA and standard biopsy**	**Strategy III PSA, MRI with targeted and standard biopsy**
**Outcomes per 100,000 men**			
Screening tests (age 55-69)	0	283 325	269 524
MRI	0	0	34 846
Biopsy	31 047	72 451	35 714
Diagnosed prostate cancer	16 542	17 401	17 003
Diagnosed prostate cancer – GG≥2	11 908	12 244	12 176
Diagnosed prostate cancer (age 55-69)	2 708	5 628	4 456
Diagnosed prostate cancer (age 55-69) – GG≥2	1 627	3 035	2 762
Over-diagnosed prostate cancer	0	858	461
Prostate cancer deaths	5 799	5 244	5 445
Life years, undiscounted	2 732 833	2 738 081	2 736 400
QALYs, undiscounted	2 186 593	2 189 487	2 188 747
QALYS, discounted at 3%	1 463 945	1 464 801	1 464 696
QALYS, discounted at 5%	1 172 350	1 172 624	1 172 682
**Costs (Million $) per 100,000 man**			
Health care perspective, undiscounted	431.6	493.8	479.5
Health care perspective, discounted at 3%	212.0	271.2	252.3
Health care perspective, discounted at 5%	138.4	193.4	174.5
Societal perspective, undiscounted	442.7	515.3	497.7
Societal perspective, discounted at 3%	220.1	288.2	266.5
Societal perspective, discounted at 5%	145.2	208.1	186.7

**Part B. Incremental cost-effectiveness ratios (ICERs) – Societal perspective, 3% discounted for QALYs and costs**				
**Strategy**	**Costs (Million $) per 100,000 men**	**QALYs per 100,000 men**	**ICERs vs. Strategy I**	**ICER Strategy II vs. III**
I No screening	220.1	1 463 945	-	-
III PSA and MRI with combined targeted and systematic biopsy	266.5	1 464 696	61 817	-
II PSA and standard biopsy	288.2	1 464 801	79 528	205 755

GG: Internal Society of Pathological Urology Grade Group; ICER: Incremental Cost-Effectiveness Ratio; MRI: Magnetic Resonance Imaging; PSA: Prostate-Specific Antigen; QALY: Quality-Adjusted Life-Year

© 2022 Hao S et al. *JAMA Oncology.*

Table S6 Summarised predictions in outcomes, costs and ICERs – Health state value set reviewed by Heijnsdijk et al (2012)

**Part A. Lifetime predictions in outcomes and costs for all strategies**			
**Lifetime predictions**	**Strategy I No screening**	**Strategy II PSA and standard biopsy**	**Strategy III PSA, MRI with targeted and standard biopsy**
**Outcomes per 100,000 men**			
Screening tests (age 55-69)	0	283 325	269 524
MRI	0	0	34 846
Biopsy	31 047	72 451	35 714
Diagnosed prostate cancer	16 542	17 401	17 003
Diagnosed prostate cancer – GG≥2	11 908	12 244	12 176
Diagnosed prostate cancer (age 55-69)	2 708	5 628	4 456
Diagnosed prostate cancer (age 55-69) – GG≥2	1 627	3 035	2 762
Over-diagnosed prostate cancer	0	858	461
Prostate cancer deaths	5 799	5 244	5 445
Life years, undiscounted	2 732 833	2 738 081	2 736 400
QALYs, undiscounted	2 184 645	2 187 675	2 186 870
QALYS, discounted at 3%	1 463 017	1 463 889	1 463 770
QALYS, discounted at 5%	1 171 755	1 172 007	1 172 068
**Costs (Million $) per 100,000 man**			
Health care perspective, undiscounted	431.6	493.8	479.5
Health care perspective, discounted at 3%	212.0	271.3	252.3
Health care perspective, discounted at 5%	138.4	193.5	174.4
Societal perspective, undiscounted	442.7	515.2	497.7
Societal perspective, discounted at 3%	220.1	288.2	266.5
Societal perspective, discounted at 5%	145.2	208.1	186.6

**Part B. Incremental cost-effectiveness ratios (ICERs) – Healthcare perspective, 3% discounted for QALYs and costs**				
**Strategy**	**Costs (Million $) per 100,000 men**	**QALYs per 100,000 men**	**ICERs vs. Strategy I**	**ICER Strategy II vs. III**
I No screening	212.0	1 463 017	-	-
III PSA and MRI with combined targeted and systematic biopsy	252.3	1 463 770	53 414	-
II PSA and standard biopsy	271.3	1 463 889	67 946	159 899

GG: Internal Society of Pathological Urology Grade Group; ICER: Incremental Cost-Effectiveness Ratio; MRI: Magnetic Resonance Imaging; PSA: Prostate-Specific Antigen; QALY: Quality-Adjusted Life-Year

© 2022 Hao S et al. *JAMA Oncology.*

Table S7 Summarised predictions in outcomes, costs and ICERs – Triennial screening

**Part A. Lifetime predictions in outcomes and costs for all strategies**			
**Lifetime predictions**	**Strategy I No screening**	**Strategy II PSA and standard biopsy**	**Strategy III PSA, MRI with targeted and standard biopsy**
**Outcomes per 100,000 men**			
Screening tests (age 55-69)	0	343 461	328 247
MRI	0	0	45 383
Biopsy	31 047	85 777	37 576
Diagnosed prostate cancer	16 542	17 638	17 138
Diagnosed prostate cancer – GG≥2	11 908	12 352	12 254
Diagnosed prostate cancer (age 55-69)	2 708	5 667	4 545
Diagnosed prostate cancer (age 55-69) – GG≥2	1 627	3 054	2 783
Over-diagnosed prostate cancer	0	1 095	595
Prostate cancer deaths	5 799	5 188	5 390
Life years, undiscounted	2 732 833	2 738 533	2 736 900
QALYs, undiscounted	2 186 593	2 189 594	2 189 007
QALYS, discounted at 3%	1 463 945	1 464 778	1 464 769
QALYS, discounted at 5%	1 172 350	1 172 573	1 172 703
**Costs (Million $) per 100,000 man**			
Health care perspective, undiscounted	431.6	514.8	491.7
Health care perspective, discounted at 3%	212.0	287.2	262.2
Health care perspective, discounted at 5%	138.4	206.9	183.1
Societal perspective, undiscounted	442.7	535.9	509.3
Societal perspective, discounted at 3%	220.1	304.0	276.0
Societal perspective, discounted at 5%	145.2	221.5	195.0

**Part B. Incremental cost-effectiveness ratios (ICERs) – Healthcare perspective, 3% discounted for QALYs and costs**				
**Strategy**	**Costs (Million $) per 100,000 men**	**QALYs per 100,000 men**	**ICERs vs. Strategy I**	**ICER Strategy II vs. III**
I No screening	212.0	1 463 945	-	-
III PSA and MRI with combined targeted and systematic biopsy	262.2	1 464 769	60 969	-
II PSA and standard biopsy	287.2	1 464 778	90 284	2 523 072

GG: Internal Society of Pathological Urology Grade Group; ICER: Incremental Cost-Effectiveness Ratio; MRI: Magnetic Resonance Imaging; PSA: Prostate-Specific Antigen; QALY: Quality-Adjusted Life-Year

© 2022 Hao S et al. *JAMA Oncology.*

Table S8 Summarised predictions in outcomes, costs and ICERs – 5-yearly screening

**Part A. Lifetime predictions in outcomes and costs for all strategies**			
**Lifetime predictions**	**Strategy I No screening**	**Strategy II PSA and standard biopsy**	**Strategy III PSA, MRI with targeted and standard biopsy**
**Outcomes per 100,000 men**			
Screening tests (age 55-69)	0	220 717	209 426
MRI	0	0	31 421
Biopsy	31 047	69 583	35 342
Diagnosed prostate cancer	16 542	17 572	17 055
Diagnosed prostate cancer – GG≥2	11 908	12 333	12 229
Diagnosed prostate cancer (age 55-69)	2 708	4 829	3 855
Diagnosed prostate cancer (age 55-69) – GG≥2	1 627	2 588	2 366
Over-diagnosed prostate cancer	0	1 029	512
Prostate cancer deaths	5 799	5 261	5 476
Life years, undiscounted	2 732 833	2 737 882	2 736 097
QALYs, undiscounted	2 186 593	2 189 303	2 188 536
QALYS, discounted at 3%	1 463 945	1 464 744	1 464 626
QALYS, discounted at 5%	1 172 350	1 172 606	1 172 655
**Costs (Million $) per 100,000 man**			
Health care perspective, undiscounted	431.6	494.1	478.7
Health care perspective, discounted at 3%	212.0	268.9	249.8
Health care perspective, discounted at 5%	138.4	190.3	171.6
Societal perspective, undiscounted	442.7	513.1	494.5
Societal perspective, discounted at 3%	220.1	283.8	262.1
Societal perspective, discounted at 5%	145.2	203.1	182.0

**Part B. Incremental cost-effectiveness ratios (ICERs) – Healthcare perspective, 3% discounted for QALYs and costs**				
**Strategy**	**Costs (Million $) per 100,000 men**	**QALYs per 100,000 men**	**ICERs vs. Strategy I**	**ICER Strategy II vs. III**
I No screening	212.0	1 463 945	-	-
III PSA and MRI with combined targeted and systematic biopsy	249.8	1 464 626	55 633	-
II PSA and standard biopsy	268.9	1 464 744	71 309	161 770

GG: Internal Society of Pathological Urology Grade Group; ICER: Incremental Cost-Effectiveness Ratio; MRI: Magnetic Resonance Imaging; PSA: Prostate-Specific Antigen; QALY: Quality-Adjusted Life-Year

© 2022 Hao S et al. *JAMA Oncology.*

Table S9 Summarised predictions in outcomes, costs and ICERs – Screening age 50-74 years

**Part A. Lifetime predictions in outcomes and costs for all strategies**			
**Lifetime predictions**	**Strategy I No screening**	**Strategy II PSA and standard biopsy**	**Strategy III PSA, MRI with targeted and standard biopsy**
**Outcomes per 100,000 men**			
Screening tests (age 50-74)	0	432 461	418 999
MRI	0	0	57 403
Biopsy	30 822	94 128	38 947
Diagnosed prostate cancer	16 416	18 300	17 636
Diagnosed prostate cancer – GG≥2	11 794	12 655	12 550
Diagnosed prostate cancer (age 50-74)	5 396	9 543	8 466
Diagnosed prostate cancer (age 50-74) – GG≥2	3 385	5 595	5 481
Over-diagnosed prostate cancer	0	1 883	1 220
Prostate cancer deaths	5 726	4 996	5 184
Life years, undiscounted	3 193 657	3 200 075	3 198 557
QALYs, undiscounted	2 570 156	2 573 232	2 572 810
QALYS, discounted at 3%	1 629 618	1 630 329	1 630 369
QALYS, discounted at 5%	1 272 517	1 272 681	1 272 801
**Costs (Million $) per 100,000 man**			
Health care perspective, undiscounted	427.4	535.2	508.8
Health care perspective, discounted at 3%	181.8	258.0	235.9
Health care perspective, discounted at 5%	108.3	168.7	150.3
Societal perspective, undiscounted	438.8	557.6	527.7
Societal perspective, discounted at 3%	189.2	273.4	248.9
Societal perspective, discounted at 5%	113.9	181.1	160.6

**Part B. Incremental cost-effectiveness ratios (ICERs) – Healthcare perspective, 3% discounted for QALYs and costs**				
**Strategy**	**Costs (Million $) per 100,000 men**	**QALYs per 100,000 men**	**ICERs vs. Strategy I**	**ICER Strategy II vs. III**
I No screening	181.8	1 629 618	-	-
III PSA and MRI with combined targeted and systematic biopsy	235.9	1 630 369	72 018	-
II PSA and standard biopsy	258.0	1 630 329	107 079	Dominated

GG: Internal Society of Pathological Urology Grade Group; ICER: Incremental Cost-Effectiveness Ratio; MRI: Magnetic Resonance Imaging; PSA: Prostate-Specific Antigen; QALY: Quality-Adjusted Life-Year

© 2022 Hao S et al. *JAMA Oncology.*

Table S10 Summarised predictions in outcomes, costs and ICERs – Adding MRI to clinical diagnosis for Strategy III

**Part A. Lifetime predictions in outcomes and costs for all strategies**			
**Lifetime predictions**	**Strategy I No screening**	**Strategy II PSA and standard biopsy**	**Strategy III PSA, MRI with targeted and standard biopsy**
**Outcomes per 100,000 men**			
Screening tests (age 55-69)	0	283 325	269 524
MRI	0	0	61 884
Biopsy	31 047	72 451	35 714
Diagnosed prostate cancer	16 542	17 401	17 003
Diagnosed prostate cancer – GG≥2	11 908	12 244	12 176
Diagnosed prostate cancer (age 55-69)	2 708	5 628	4 456
Diagnosed prostate cancer (age 55-69) – GG≥2	1 627	3 035	2 762
Over-diagnosed prostate cancer	0	858	461
Prostate cancer deaths	5 799	5 244	5 445
Life years, undiscounted	2 732 833	2 738 081	2 736 400
QALYs, undiscounted	2 186 593	2 189 487	2 188 747
QALYS, discounted at 3%	1 463 945	1 464 801	1 464 696
QALYS, discounted at 5%	1 172 350	1 172 624	1 172 682
**Costs (Million $) per 100,000 man**			
Health care perspective, undiscounted	431.6	493.8	494.2
Health care perspective, discounted at 3%	212.0	271.2	259.7
Health care perspective, discounted at 5%	138.4	193.4	179.3
Societal perspective, undiscounted	442.7	515.3	512.5
Societal perspective, discounted at 3%	220.1	288.2	273.9
Societal perspective, discounted at 5%	145.2	208.1	191.5

**Part B. Incremental cost-effectiveness ratios (ICERs) – Healthcare perspective, 3% discounted for QALYs and costs**				
**Strategy**	**Costs (Million $) per 100,000 men**	**QALYs per 100,000 men**	**ICERs vs. Strategy I**	**ICER Strategy II vs. III**
I No screening	212.0	1 463 945	-	-
III PSA and MRI with combined targeted and systematic biopsy	259.7	1 464 696	63 546	-
II PSA and standard biopsy	271.2	1 464 801	69 254	109 939

GG: Internal Society of Pathological Urology Grade Group; ICER: Incremental Cost-Effectiveness Ratio; MRI: Magnetic Resonance Imaging; PSA: Prostate-Specific Antigen; QALY: Quality-Adjusted Life-Year

Table S11 Summarised predictions in outcomes, costs and ICERs – Reduce biopsy cost by 50%

**Part A. Lifetime predictions in outcomes and costs for all strategies**			
**Lifetime predictions**	**Strategy I No screening**	**Strategy II PSA and standard biopsy**	**Strategy III PSA, MRI with targeted and standard biopsy**
**Outcomes per 100,000 men**			
Screening tests (age 55-69)	0	283 325	269 524
MRI	0	0	34 846
Biopsy	31 047	72 451	35 714
Diagnosed prostate cancer	16 542	17 401	17 003
Diagnosed prostate cancer – GG≥2	11 908	12 244	12 176
Diagnosed prostate cancer (age 55-69)	2 708	5 628	4 456
Diagnosed prostate cancer (age 55-69) – GG≥2	1 627	3 035	2 762
Over-diagnosed prostate cancer	0	858	461
Prostate cancer deaths	5 799	5 244	5 445
Life years, undiscounted	2 732 833	2 738 081	2 736 400
QALYs, undiscounted	2 186 593	2 189 487	2 188 747
QALYS, discounted at 3%	1 463 945	1 464 801	1 464 696
QALYS, discounted at 5%	1 172 350	1 172 624	1 172 682
**Costs (Million $) per 100,000 man**			
Health care perspective, undiscounted	415.8	461.4	457.4
Health care perspective, discounted at 3%	204.1	249.9	240.3
Health care perspective, discounted at 5%	133.2	176.6	166.1
Societal perspective, undiscounted	426.9	482.8	475.6
Societal perspective, discounted at 3%	212.2	266.9	254.5
Societal perspective, discounted at 5%	139.9	191.2	178.2

**Part B. Incremental cost-effectiveness ratios (ICERs) – Healthcare perspective, 3% discounted for QALYs and costs**				
**Strategy**	**Costs (Million $) per 100,000 men**	**QALYs per 100,000 men**	**ICERs vs. Strategy I**	**ICER Strategy II vs. III**
I No screening	204.1	1 463 945	-	-
III PSA and MRI with combined targeted and systematic biopsy	240.3	1 464 696	48 322	-
II PSA and standard biopsy	249.9	1 464 801	53 598	91 201

GG: Internal Society of Pathological Urology Grade Group; ICER: Incremental Cost-Effectiveness Ratio; MRI: Magnetic Resonance Imaging; PSA: Prostate-Specific Antigen; QALY: Quality-Adjusted Life-Year

© 2022 Hao S et al. *JAMA Oncology.*

Table S12 Summarised predictions in outcomes, costs and ICERs – Increase biopsy cost by 50%

**Part A. Lifetime predictions in outcomes and costs for all strategies**			
**Lifetime predictions**	**Strategy I No screening**	**Strategy II PSA and standard biopsy**	**Strategy III PSA, MRI with targeted and standard biopsy**
**Outcomes per 100,000 men**			
Screening tests (age 55-69)	0	283 325	269 524
MRI	0	0	34 846
Biopsy	31 047	72 451	35 714
Diagnosed prostate cancer	16 542	17 401	17 003
Diagnosed prostate cancer – GG≥2	11 908	12 244	12 176
Diagnosed prostate cancer (age 55-69)	2 708	5 628	4 456
Diagnosed prostate cancer (age 55-69) – GG≥2	1 627	3 035	2 762
Over-diagnosed prostate cancer	0	858	461
Prostate cancer deaths	5 799	5 244	5 445
Life years, undiscounted	2 732 833	2 738 081	2 736 400
QALYs, undiscounted	2 186 593	2 189 487	2 188 747
QALYS, discounted at 3%	1 463 945	1 464 801	1 464 696
QALYS, discounted at 5%	1 172 350	1 172 624	1 172 682
**Costs (Million $) per 100,000 man**			
Health care perspective, undiscounted	447.4	526.3	494.2
Health care perspective, discounted at 3%	219.9	292.5	260.8
Health care perspective, discounted at 5%	143.7	210.3	180.8
Societal perspective, undiscounted	458.5	547.8	512.5
Societal perspective, discounted at 3%	228.0	309.5	275.0
Societal perspective, discounted at 5%	150.4	224.9	192.9

**Part B. Incremental cost-effectiveness ratios (ICERs) – Healthcare perspective, 3% discounted for QALYs and costs**				
**Strategy**	**Costs (Million $) per 100,000 men**	**QALYs per 100,000 men**	**ICERs vs. Strategy I**	**ICER Strategy II vs. III**
I No screening	219.9	1 463 945	-	-
III PSA and MRI with combined targeted and systematic biopsy	260.8	1 464 696	54 584	-
II PSA and standard biopsy	292.5	1 464 801	84 909	301 054

GG: Internal Society of Pathological Urology Grade Group; ICER: Incremental Cost-Effectiveness Ratio; MRI: Magnetic Resonance Imaging; PSA: Prostate-Specific Antigen; QALY: Quality-Adjusted Life-Year

Table S13 Summarised predictions in outcomes, costs and ICERs – Reduce MRI cost by 50%

**Part A. Lifetime predictions in outcomes and costs for all strategies**			
**Lifetime predictions**	**Strategy I No screening**	**Strategy II PSA and standard biopsy**	**Strategy III PSA, MRI with targeted and standard biopsy**
**Outcomes per 100,000 men**			
Screening tests (age 55-69)	0	283 325	269 524
MRI	0	0	34 846
Biopsy	31 047	72 451	35 714
Diagnosed prostate cancer	16 542	17 401	17 003
Diagnosed prostate cancer – GG≥2	11 908	12 244	12 176
Diagnosed prostate cancer (age 55-69)	2 708	5 628	4 456
Diagnosed prostate cancer (age 55-69) – GG≥2	1 627	3 035	2 762
Over-diagnosed prostate cancer	0	858	461
Prostate cancer deaths	5 799	5 244	5 445
Life years, undiscounted	2 732 833	2 738 081	2 736 400
QALYs, undiscounted	2 186 593	2 189 487	2 188 747
QALYS, discounted at 3%	1 463 945	1 464 801	1 464 696
QALYS, discounted at 5%	1 172 350	1 172 624	1 172 682
**Costs (Million $) per 100,000 man**			
Health care perspective, undiscounted	431.6	493.8	468.5
Health care perspective, discounted at 3%	212.0	271.2	245.1
Health care perspective, discounted at 5%	138.4	193.4	168.8
Societal perspective, undiscounted	442.7	515.3	486.8
Societal perspective, discounted at 3%	220.1	288.2	259.3
Societal perspective, discounted at 5%	145.2	208.1	181.0

**Part B. Incremental cost-effectiveness ratios (ICERs) – Healthcare perspective, 3% discounted for QALYs and costs**				
**Strategy**	**Costs (Million $) per 100,000 men**	**QALYs per 100,000 men**	**ICERs vs. Strategy I**	**ICER Strategy II vs. III**
I No screening	212.0	1 463 945	-	-
III PSA and MRI with combined targeted and systematic biopsy	245.1	1 464 696	44 148	-
II PSA and standard biopsy	271.2	1 464 801	69 254	248 195

GG: Internal Society of Pathological Urology Grade Group; ICER: Incremental Cost-Effectiveness Ratio; MRI: Magnetic Resonance Imaging; PSA: Prostate-Specific Antigen; QALY: Quality-Adjusted Life-Year

© 2022 Hao S et al. *JAMA Oncology.*

Table S14 Summarised predictions in outcomes, costs and ICERs – Increase MRI cost by 50%

**Part A. Lifetime predictions in outcomes and costs for all strategies**			
**Lifetime predictions**	**Strategy I No screening**	**Strategy II PSA and standard biopsy**	**Strategy III PSA, MRI with targeted and standard biopsy**
**Outcomes per 100,000 men**			
Screening tests (age 55-69)	0	283 325	269 524
MRI	0	0	34 846
Biopsy	31 047	72 451	35 714
Diagnosed prostate cancer	16 542	17 401	17 003
Diagnosed prostate cancer – GG≥2	11 908	12 244	12 176
Diagnosed prostate cancer (age 55-69)	2 708	5 628	4 456
Diagnosed prostate cancer (age 55-69) – GG≥2	1 627	3 035	2 762
Over-diagnosed prostate cancer	0	858	461
Prostate cancer deaths	5 799	5 244	5 445
Life years, undiscounted	2 732 833	2 738 081	2 736 400
QALYs, undiscounted	2 186 593	2 189 487	2 188 747
QALYS, discounted at 3%	1 463 945	1 464 801	1 464 696
QALYS, discounted at 5%	1 172 350	1 172 624	1 172 682
**Costs (Million $) per 100,000 man**			
Health care perspective, undiscounted	431.6	493.8	490.0
Health care perspective, discounted at 3%	212.0	271.2	259.3
Health care perspective, discounted at 5%	138.4	193.4	180.1
Societal perspective, undiscounted	442.7	515.3	508.2
Societal perspective, discounted at 3%	220.1	288.2	273.5
Societal perspective, discounted at 5%	145.2	208.1	192.2

**Part B. Incremental cost-effectiveness ratios (ICERs) – Healthcare perspective, 3% discounted for QALYs and costs**				
**Strategy**	**Costs (Million $) per 100,000 men**	**QALYs per 100,000 men**	**ICERs vs. Strategy I**	**ICER Strategy II vs. III**
I No screening	212.0	1 463 945	-	-
III PSA and MRI with combined targeted and systematic biopsy	259.3	1 464 696	63 036	-
II PSA and standard biopsy	271.2	1 464 801	69 254	113 569

GG: Internal Society of Pathological Urology Grade Group; ICER: Incremental Cost-Effectiveness Ratio; MRI: Magnetic Resonance Imaging; PSA: Prostate-Specific Antigen; QALY: Quality-Adjusted Life-Year

© 2022 Hao S et al. *JAMA Oncology.*

Table S15 Summarised predictions in outcomes, costs and ICERs – Replacing combined biopsies by targeted biopsy for Strategy III

**Part A. Lifetime predictions in outcomes and costs for all strategies**			
**Lifetime predictions**	**Strategy I No screening**	**Strategy II PSA and standard biopsy**	**Strategy III PSA, MRI with targeted and standard biopsy**
**Outcomes per 100,000 men**			
Screening tests (age 55-69)	0	283 325	270 130
MRI	0	0	35 358
Biopsy	31 047	72 451	36 493
Diagnosed prostate cancer	16 542	17 401	16 917
Diagnosed prostate cancer – GG≥2	11 908	12 244	12 171
Diagnosed prostate cancer (age 55-69)	2 708	5 628	4 184
Diagnosed prostate cancer (age 55-69) – GG≥2	1 627	3 035	2 744
Over-diagnosed prostate cancer	0	858	374
Prostate cancer deaths	5 799	5 244	5 497
Life years, undiscounted	2 732 833	2 738 081	2 735 973
QALYs, undiscounted	2 186 593	2 189 487	2 188 495
QALYS, discounted at 3%	1 463 945	1 464 801	1 464 623
QALYS, discounted at 5%	1 172 350	1 172 624	1 172 660
**Costs (Million $) per 100,000 man**			
Health care perspective, undiscounted	431.6	493.8	474.6
Health care perspective, discounted at 3%	212.0	271.2	248.5
Health care perspective, discounted at 5%	138.4	193.4	171.2
Societal perspective, undiscounted	442.7	515.3	492.0
Societal perspective, discounted at 3%	220.1	288.2	262.0
Societal perspective, discounted at 5%	145.2	208.1	182.7

**Part B. Incremental cost-effectiveness ratios (ICERs) – Healthcare perspective, 3% discounted for QALYs and costs**				
**Strategy**	**Costs (Million $) per 100,000 men**	**QALYs per 100,000 men**	**ICERs vs. Strategy I**	**ICER Strategy II vs. III**
I No screening	212.0	1 463 945	-	-
III PSA and MRI with combined targeted and systematic biopsy	248.5	1 464 623	53 866	-
II PSA and standard biopsy	271.2	1 464 801	69 254	128 026

GG: Internal Society of Pathological Urology Grade Group; ICER: Incremental Cost-Effectiveness Ratio; MRI: Magnetic Resonance Imaging; PSA: Prostate-Specific Antigen; QALY: Quality-Adjusted Life-Year

© 2022 Hao S et al. *JAMA Oncology.*

Table S16 Summarised predictions in outcomes, costs and ICERs – Stratified rescreening

**Part A. Lifetime predictions in outcomes and costs for all strategies**			
**Lifetime predictions**	**Strategy I No screening**	**Strategy II PSA and standard biopsy**	**Strategy III PSA, MRI with targeted and standard biopsy**
**Outcomes per 100,000 men**			
Screening tests (age 55-69)	0	283 325	308 800
MRI	0	0	64 226
Biopsy	31 047	72 451	40 680
Diagnosed prostate cancer	16 542	17 401	17 296
Diagnosed prostate cancer – GG≥2	11 908	12 244	12 321
Diagnosed prostate cancer (age 55-69)	2 708	5 628	5 370
Diagnosed prostate cancer (age 55-69) – GG≥2	1 627	3 035	3 288
Over-diagnosed prostate cancer	0	858	753
Prostate cancer deaths	5 799	5 244	5 301
Life years, undiscounted	2 732 833	2 738 081	2 737 641
QALYs, undiscounted	2 186 593	2 189 487	2 189 385
QALYS, discounted at 3%	1 463 945	1 464 801	1 464 866
QALYS, discounted at 5%	1 172 350	1 172 624	1 172 726
**Costs (Million $) per 100,000 man**			
Health care perspective, undiscounted	431.6	493.8	503.0
Health care perspective, discounted at 3%	212.0	271.2	272.3
Health care perspective, discounted at 5%	138.4	193.4	192.1
Societal perspective, undiscounted	442.7	515.3	522.8
Societal perspective, discounted at 3%	220.1	288.2	287.8
Societal perspective, discounted at 5%	145.2	208.1	205.4

**Part B. Incremental cost-effectiveness ratios (ICERs) – Healthcare perspective, 3% discounted for QALYs and costs**				
**Strategy**	**Costs (Million $) per 100,000 men**	**QALYs per 100,000 men**	**ICERs vs. Strategy I**	**ICER Strategy II vs. III**
I No screening	212.0	1 463 945	-	-
III PSA and MRI with combined targeted and systematic biopsy	271.2	1 464 801	69 254	-
II PSA and standard biopsy	272.3	1 464 866	65 488	16 184

GG: Internal Society of Pathological Urology Grade Group; ICER: Incremental Cost-Effectiveness Ratio; MRI: Magnetic Resonance Imaging; PSA: Prostate-Specific Antigen; QALY: Quality-Adjusted Life-Year

© 2022 Hao S et al. *JAMA Oncology.*

Table S17 Summarised predictions in outcomes, costs and ICERs – 80% attendance of rescreening

**Part A. Lifetime predictions in outcomes and costs for all strategies**			
**Lifetime predictions**	**Strategy I No screening**	**Strategy II PSA and standard biopsy**	**Strategy III PSA, MRI with targeted and standard biopsy**
**Outcomes per 100,000 men**			
Screening tests (age 55-69)	0	226 232	215 849
MRI	0	0	24 970
Biopsy	31 047	60 889	34 393
Diagnosed prostate cancer	16 542	17 121	16 850
Diagnosed prostate cancer – GG≥2	11 908	12 133	12 087
Diagnosed prostate cancer (age 55-69)	2 708	4 694	3 869
Diagnosed prostate cancer (age 55-69) – GG≥2	1 627	2 563	2 375
Over-diagnosed prostate cancer	0	579	308
Prostate cancer deaths	5 799	5 391	5 545
Life years, undiscounted	2 732 833	2 736 832	2 735 481
QALYs, undiscounted	2 186 593	2 188 881	2 188 231
QALYS, discounted at 3%	1 463 945	1 464 657	1 464 532
QALYS, discounted at 5%	1 172 350	1 172 601	1 172 618
**Costs (Million $) per 100,000 man**			
Health care perspective, undiscounted	431.6	475.9	469.4
Health care perspective, discounted at 3%	212.0	255.5	243.2
Health care perspective, discounted at 5%	138.4	179.6	166.3
Societal perspective, undiscounted	442.7	495.0	486.0
Societal perspective, discounted at 3%	220.1	270.6	256.0
Societal perspective, discounted at 5%	145.2	192.7	177.3

**Part B. Incremental cost-effectiveness ratios (ICERs) – Healthcare perspective, 3% discounted for QALYs and costs**				
**Strategy**	**Costs (Million $) per 100,000 men**	**QALYs per 100,000 men**	**ICERs vs. Strategy I**	**ICER Strategy II vs. III**
I No screening	212.0	1 463 945	-	-
III PSA and MRI with combined targeted and systematic biopsy	243.2	1 464 532	53 156	-
II PSA and standard biopsy	255.5	1 464 657	61 206	Dominated

GG: Internal Society of Pathological Urology Grade Group; ICER: Incremental Cost-Effectiveness Ratio; MRI: Magnetic Resonance Imaging; PSA: Prostate-Specific Antigen; QALY: Quality-Adjusted Life-Year

© 2022 Hao S et al. *JAMA Oncology.*

Table S18 Summarised predictions in outcomes, costs and ICERs – Healthcare perspective, discount rate 0%

**Part B. Incremental cost-effectiveness ratios (ICERs) – Societal perspective, undiscounted for QALYs and costs**				
**Strategy**	**Costs (Million $) per 100,000 men**	**QALYs per 100,000 men**	**ICERs vs. Strategy I**	**ICER Strategy II vs. III**
I No screening	431.6	2 186 593	-	-
III PSA and MRI with combined targeted and systematic biopsy	479.5	2 188 747	22 246	-
II PSA and standard biopsy	493.8	2 189 487	21 509	19 364

ICER: Incremental Cost-Effectiveness Ratio; MRI: Magnetic Resonance Imaging; PSA: Prostate-Specific Antigen; QALY: Quality-Adjusted Life-Year

Table S19 Summarised predictions in outcomes, costs and ICERs – Healthcare perspective, costs and QALYs discounted at 5%

**Part B. Incremental cost-effectiveness ratios (ICERs) – Societal perspective, undiscounted for QALYs and costs**				
**Strategy**	**Costs (Million $) per 100,000 men**	**QALYs per 100,000 men**	**ICERs vs. Strategy I**	**ICER Strategy II vs. III**
I No screening	138.4	1 172 350	-	-
III PSA and MRI with combined targeted and systematic biopsy	174.5	1 172 682	108 588	-
II PSA and standard biopsy	193.4	1 172 624	200 739	Dominated

ICER: Incremental Cost-Effectiveness Ratio; MRI: Magnetic Resonance Imaging; PSA: Prostate-Specific Antigen; QALY: Quality-Adjusted Life-Year

Table S20 Summarised predictions in outcomes, costs and ICERs – Healthcare perspective, costs discounted at 3%, QALYs undiscounted

**Part B. Incremental cost-effectiveness ratios (ICERs) – Societal perspective, undiscounted for QALYs and costs**				
**Strategy**	**Costs (Million $) per 100,000 men**	**QALYs per 100,000 men**	**ICERs vs. Strategy I**	**ICER Strategy II vs. III**
I No screening	212.0	2 186 593	-	-
III PSA and MRI with combined targeted and systematic biopsy	252.3	2 188 747	18 720	-
II PSA and standard biopsy	271.2	2 189 487	20 477	25 589

ICER: Incremental Cost-Effectiveness Ratio; MRI: Magnetic Resonance Imaging; PSA: Prostate-Specific Antigen; QALY: Quality-Adjusted Life-Year

© 2022 Hao S et al. *JAMA Oncology.*

Table S21 Summarised predictions in outcomes, costs and ICERs – Base case, discounted from age 50 years

**Part A. Lifetime predictions in outcomes and costs for all strategies**			
**Lifetime predictions**	**Strategy I No screening**	**Strategy II PSA and standard biopsy**	**Strategy III PSA, MRI with targeted and standard biopsy**
**Outcomes per 100,000 men**			
Screening tests (age 55-69)	0	279 573	265 955
MRI	0	0	34 385
Biopsy	30 822	71 678	35 428
Diagnosed prostate cancer	16 416	17 263	16 871
Diagnosed prostate cancer – GG≥2	11 794	12 126	12 058
Diagnosed prostate cancer (age 55-69)	5 396	5 553	4 397
Diagnosed prostate cancer (age 55-69) – GG≥2	3 385	2 994	2 726
Over-diagnosed prostate cancer	0	847	455
Prostate cancer deaths	5 726	5 178	5 377
Life years, undiscounted	3 193 657	3 198 835	3 197 176
QALYs, undiscounted	2 570 156	2 573 011	2 572 281
QALYS, discounted at 3%	1 629 618	1 630 346	1 630 257
QALYS, discounted at 5%	1 272 517	1 272 728	1 272 773
**Costs (Million $) per 100,000 man**			
Health care perspective, undiscounted	427.4	488.8	474.7
Health care perspective, discounted at 3%	181.8	232.3	216.2
Health care perspective, discounted at 5%	108.3	150.9	136.2
Societal perspective, undiscounted	438.8	510.4	493.1
Societal perspective, discounted at 3%	189.2	247.1	228.7
Societal perspective, discounted at 5%	113.9	162.6	146.0

**Part B. Incremental cost-effectiveness ratios (ICERs) – Healthcare perspective, 3% discounted for QALYs and costs**				
**Strategy**	**Costs (Million $) per 100,000 men**	**QALYs per 100,000 men**	**ICERs vs. Strategy I**	**ICER Strategy II vs. III**
I No screening	181.8	1 629 618	-	-
III PSA and MRI with combined targeted and systematic biopsy	216.2	1 630 257	53 765	-
II PSA and standard biopsy	232.3	1 630 346	69 254	Dominated

GG: Internal Society of Pathological Urology Grade Group; ICER: Incremental Cost-Effectiveness Ratio; MRI: Magnetic Resonance Imaging; PSA: Prostate-Specific Antigen; QALY: Quality-Adjusted Life-Year

© 2022 Hao S et al. *JAMA Oncology.*

Figure S2 Cost-effectiveness plane of the probabilistic sensitivity analysis

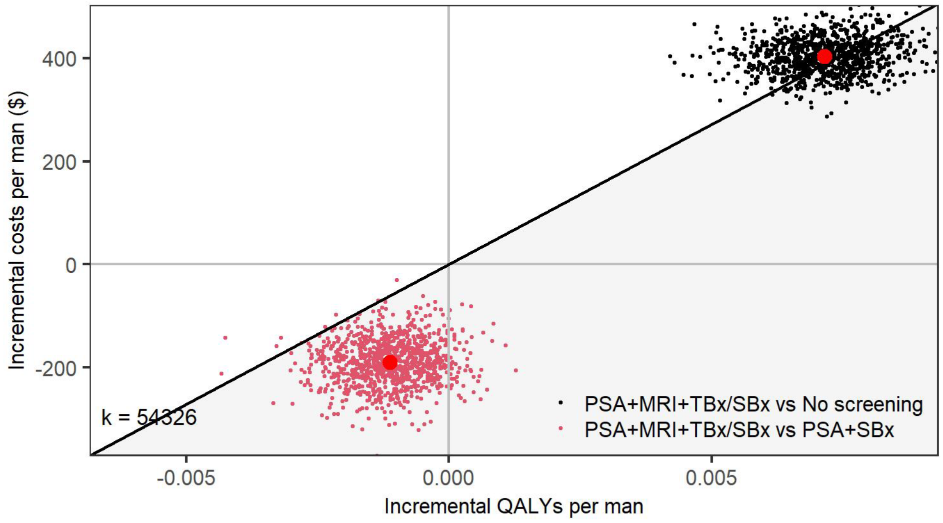

The cost-effectiveness plane of the probabilistic sensitivity analysis shows robust results of ICERs for the comparisons. The scatter plot plots each simulated estimate of the incremental costs and QALYs for each comparison. Compared with no screening, the MRI-based screening with the combined TBx/SBx was expected to have higher costs and higher QALYs. Approximately 40% of the points were under the nominal cost-effectiveness threshold of USD 54,326 (500 000 SEK). Compared with PSA based screening using SBx, the MRI-based screening were expected to have lower costs and similar QALYs.

MRI: Magnetic Resonance Imaging; PSA: Prostate-Specific Antigen; QALYs: Quality-Adjusted Life-Years; SEK: Swedish Krona; SBx: Standard Biopsy; TBx/SBx: Combined Targeted and Standard Biopsy; USD: United States Dollar

© 2022 Hao S et al. *JAMA Oncology.*

References

1. van Buuren S, Groothuis-Oudshoorn K. mice: Multivariate Imputation by Chained Equations in R. J Stat Softw 2011;45(3):1-67. (In English) (<Go to ISI>://WOS:000298032500001).
2. Eklund M, Jaderling F, Discacciati A, et al. MRI-Targeted or Standard Biopsy in Prostate Cancer Screening. The New England journal of medicine 2021. DOI: 10.1056/NEJMoa2100852.
3. Socialstyrelsen. Öppna jämförelser 2014 Hälso- och sjukvårdHälso- och sjukvård. Jämförelser mellan landsting. Del 1. Övergripande indikatorer. 2014.
4. Socialstyrelsen. Öppna jämförelser 2015 Hälso- och sjukvård. Övergripande indikatorer. 2015.
5. SödraRegionvårdsnämnden. Regionala priser och ersättningar för södra sjukvårdsregionen 2015. 2015.
6. SödraRegionvårdsnämnden. Regionala priser och ersättningar för södra sjukvårdsregionen 2016. 2016.
7. Socialstyrelsen. Screening för prostatacancer med PSA-prov. Hälsoekonomisk analys Bilaga. February 13, 2018.
8. SLL. Avtal med Karolinska sjukhuset avseende tjänster inom klinisk laboratoriemedicin i sydöstra länet HSN 2018-0963. Stockholm: 2018.
9. SLL. Vårdval specialiserad urologi. 2020-12-01 2020. (https://vardgivarguiden.se/globalassets/avtal/vardavtal/vardval-stockholm/urologi/ffu-urologi.pdf?IsPdf=true).
10. Socialstyrelsen. Hälsoekonomiskt underlag. Nationella riktlinjer för prostatacancer 2014. 2014. (https://www.socialstyrelsen.se/globalassets/sharepoint-dokument/artikelkatalog/nationella-riktlinjer/nr-cancer-halsoekonomiskt-underlag-prostatacancer.pdf).
11. Hao S, Östensson E, Eklund M, et al. The economic burden of prostate cancer – a Swedish prevalence-based register study. BMC Health Services Research 2020;20(1):448. DOI: 10.1186/s12913-020-05265-8.
12. Heijnsdijk EA, de Carvalho TM, Auvinen A, et al. Cost-effectiveness of prostate cancer screening: a simulation study based on ERSPC data. Journal of the National Cancer Institute 2015;107(1):366. DOI: 10.1093/jnci/dju366.
13. Statistikmyndigheten. Population Aged 15-74 (LFS) by Sex, Age and Labour Status. Year 1970 - 2020. January 28, 2021 ed. Örebro: Statistikmyndigheten; 2021.
14. Statistikmyndigheten. Average basic salary, monthly salary and women´s salary as a percentage of men´s salary by sector, occupation (SSYK 2012), sex and educational level (SUN) . Year 2014 - 2020. June 22, 2021 ed. Örebro: Statistikmyndigheten; 2021.
15. Armelius H. Sociala avgifter över tid - Avtalade och lagstadgade avgifter - arbetare. ekonomifakta. 2021-08-16 (https://www.ekonomifakta.se/Fakta/Skatter/Skatt-pa-arbete/Sociala-avgifter-over-tid/).
16. Statistikmyndigheten. CPI, Fixed Index Numbers (1980=100). Statistikmyndigheten. 2021-10-14 (https://www.scb.se/en/finding-statistics/statistics-by-subject-area/prices-and-consumption/consumer-price-index/consumer-price-index-cpi/pong/tables-and-graphs/consumer-price-index-cpi/cpi-fixed-index-numbers-1980100/).
17. Riksbanken. Annual average exchange rates. Stockholm: Swedish National Bank; 2022.
18. FASS. Farmaceutiska Specialiteter i Sverige. 2022.
19. Bratt O. Prostatacancer. Internetmedicin, July 2, 2021 2021. (https://www.internetmedicin.se/behandlingsoversikter/kirurgi/prostatacancer/).
20. E-Hälsömyndigheten. Statistik och läkemedelsförsäljning. (https://www.ehalsomyndigheten.se/statistik-och-lakemedelsforsaljning/).
21. NPCR. RATTEN - Interactive On Line Report from NPCR. 2021-08-24 ed: National Prostate Cancer Register of Sweden; 2021.
22. Hao S, Karlsson A, Heintz E, Elfström KM, Nordström T, Clements M. Cost-Effectiveness of Magnetic Resonance Imaging in Prostate Cancer Screening: A Microsimulation Study. Value in Health 2021.
23. Magnus A, Isaranuwatchai W, Mihalopoulos C, Brown V, Carter R. A Systematic Review and Meta-Analysis of Prostate Cancer Utility Values of Patients and Partners Between 2007 and 2016. MDM Policy Pract 2019;4(1):2381468319852332. DOI: 10.1177/2381468319852332.
24. palliativregistret S. The Swedish Register of Palliative Care (SRPC). (www.palliativregistret.se).
25. Burström K, Johannesson M, Diderichsen F. Swedish population health-related quality of life results using the EQ-5D. Qual Life Res 2001;10(7):621-35. (In eng). DOI: 10.1023/a:1013171831202.

© 2022 Hao S et al. *JAMA Oncology.*
